# The polygenic implication of clopidogrel responsiveness: Insights from platelet reactivity analysis and next-generation sequencing

**DOI:** 10.1371/journal.pone.0306445

**Published:** 2024-07-11

**Authors:** Omar Echeverría, Mariana Angulo-Aguado, Ricardo Vela, Carlos Calderón-Ospina, Katherine Parra, Nora Contreras, Adrien Morel, Rodrigo Cabrera, Carlos Restrepo, Carolina Ramírez-Santana, Oscar Ortega-Recalde, Manuel Eduardo Rojas-Quintana, Luisa Murcia, Cristian Camilo Gaviria-Sabogal, Nattaly Valero, Dora Janeth Fonseca-Mendoza

**Affiliations:** 1 School of Medicine and Health Sciences, Center for Research in Genetics and Genomics (CIGGUR), Institute of Translational Medicine (IMT), Universidad Del Rosario, Bogotá D.C., Colombia; 2 Hospital Universitario Mayor—Méderi—Universidad del Rosario, Bogotá D.C., Colombia; 3 Center for Autoimmune Diseases Research (CREA), School of Medicine and Health Sciences, Universidad del Rosario, Bogotá D.C., Colombia; 4 Departamento de Morfología, Facultad de Medicina, Universidad Nacional de Colombia, Bogotá D.C., Colombia; 5 Division of Rheumatology, Allergy and Clinical Immunology, University of California, Davis, CA, United States of America; Universiti Sains Malaysia, MALAYSIA

## Abstract

Clopidogrel is widely used worldwide as an antiplatelet therapy in patients with acute coronary disease. Genetic factors influence interindividual variability in response. Some studies have explored the polygenic contributions in the drug response, generating pharmacogenomic risk scores (PgxPRS). Importantly, these factors are less explored in underrepresented populations, such as Latin-American countries. Identifying patients at risk of high-on-treatment platelet reactivity (HTPR) is highly valuable in translational medicine. In this study we used a custom next-generation sequencing (NGS) panel composed of 91 single nucleotide polymorphisms (SNPs) and 28 genes related to clopidogrel metabolism, to analyze 70 patients with platelet reactivity values, assessed through closure time (CT). Our results demonstrated the association of SNPs with HTPR and non-HTPR, revealing the strongest associations with rs2286823 (OR: 5,0; 95% CI: 1,02–24,48; p: 0,03), rs2032582 (OR: 4,41; 95% CI: 1,20–16,12; p: 0,019), and rs1045642 (OR: 3,38; 95% CI: 0,96–11,9; p: 0,05). Bivariate regression analysis demonstrated the significant association of several SNPs with the CT value, a “surrogate” biomarker of clopidogrel response. Exploratory results from the LASSO regression model showed a high discriminatory capacity between HTPR and non-HTPR patients (AUC: 0,955), and the generated PgxPRS demonstrated a significant negative association between the risk score, CT value, and the condition of HTPR and non-HTPR. To our knowledge, our study addresses for the first time the analysis of the polygenic contribution in platelet reactivity using NGS and establishes PgxPRS derived from the LASSO model. Our results demonstrate the polygenic implication of clopidogrel response and offer insights applicable to the translational medicine of antiplatelet therapy in an understudied population.

## Introduction

Clopidogrel is an antiplatelet therapy widely prescribed worldwide for antiplatelet therapy in the prevention of atherothrombotic events in patients with coronary heart disease. Clopidogrel is a prodrug that requires biotransformation into an active metabolite by cytochrome P-450 (CYP-450) enzymes. The P2Y12 receptor is inhibited by the active metabolite of clopidogrel, playing a role in adenosine diphosphate (ADP)-induced platelet activation and aggregation [1]. Clopidogrel response can be evaluated by determining platelet reactivity through ADP-induced light transmittance aggregometry (LTA), the Verify Now P2Y12 assay, and the INNOVANCE PFA-200 system, which can discriminate between patients with high on-treatment platelet reactivity (HTPR) or low on-treatment platelet reactivity (LTPR) [2]. Importantly, there is significant interindividual variability in the response, with some patients experiencing HTPR and an increased risk of adverse effects [3].

The variability in clopidogrel efficacy has been linked to non-genetic factors such as age, sex, comorbidities, and drug-drug interactions, among others. Platelet response to clopidogrel is highly heritable h(2) = 0,73 and has been associated with molecular variants in genes related to drug pharmacokinetics, and pharmacodynamics [4]. The primary worldwide genetic determinant is the single nucleotide polymorphism (SNP) CYP2C19*2 (rs4244285). Individuals carrying this loss-of-function allele convert clopidogrel to its active metabolite less efficiently, leading to reduced platelet inhibition. This variant, however, explains only about 12% of the variation in HTPR in Europeans, leaving a significant percentage of the variability in the response to clopidogrel unexplained [5].

Numerous studies have been conducted to identify genetic determinants of clopidogrel response, primarily through genome-wide association studies (GWAS) and case-control studies with specific variants, proposing candidate genes such as *CYP2C19*, *CES1*, *CYP2B6*, *CYP2C9*, *P2Y12*, *PON1*, among others [5–7]. These findings have demonstrated the polygenic effect on clopidogrel response raising proposals for pharmacogenomic polygenic risk scores (PgxRS) applicable to identifying patients with HTPR [5, 8]. Polygenic risk scores represent effective approaches to improve the precision of risk-based screening programs for disease, but they have been less explored in pharmacogenomics [9]. Most studies to date have predominantly focused on individuals of European ancestry, and, except for analyses conducted in Caribbean Hispanic populations, there remains a notable knowledge gap for the Latin-American population, including Colombia [10].

Pharmacogenomic analyses to identify molecular variants of interest have primarily been developed by analyzing common variants through SNP microarrays. However, recently, the implementation of next-generation sequencing (NGS) has demonstrated high versatility in identifying both rare and common variants that explain the missing heritability associated with drug response phenotypes [11]. In this study, we employed an innovative strategy by developing a custom panel for the analysis of 91 SNPs (derived from previous GWAS studies) and 28 genes associated with clopidogrel pharmacokinetics and pharmacodynamics through NGS.

To the best of our knowledge, our study is the first to utilize the LASSO regression model and construct a PgxPRS for discriminating patients with an inadequate response to clopidogrel, determined through platelet reactivity measured as closure time (CT). The exploratory LASSO model demonstrated a high discriminatory power (Area Under the Curve- AUC:0,955), and the PgxPRS score was statistically associated with clopidogrel response. Our results hold significant implications for translational medicine in the field of antiplatelet therapy.

## Materials and methods

### Patients

This study included 70 patients admitted to the Hospital Universitario Mayor-Méderi with a diagnosis of acute coronary syndrome and treated with a loading dose of 300 mg/75 mg of clopidogrel for at least seven consecutive days. Eligible patients, aged 18 years or older, were invited to participate in the study and provided written informed consent. Exclusion criteria comprised individuals receiving oral anticoagulants and glycoprotein IIa/IIb receptor inhibitors, hematocrit values below 25% or above 52%, platelet count <100x10^9^/L, creatinine >15 mg/dL, and clinical evidence of hepatic damage or platelet dysfunction.

Non-genetic variables, such as sex, body mass index, comorbidities (diabetes, hypertension, and dyslipidemia), smoking, alcohol consumption, type of acute coronary syndrome, type of intervention, stent usage, family history, use of CYP2C19 inhibitors, and inducers, among others, were collected. The 70 patients in our study were enrolled from a previous study described by Angulo-Aguado et al. in 2021 [12]. For the current research, access was granted to DNA samples stored as of March 30, 2022. Throughout the project development, the anonymity of the samples was maintained, adhering to the initially assigned coding.

All experimental procedures were approved by the ethics committee of the Universidad del Rosario and followed the Helsinki Declaration guidelines (Approval DVO005 1785-CV1486).

### Platelet function test

As previously described [12], platelet function was evaluated using the INNOVANCE PFA-200 P2Y system (Siemens Healthcare). Blood samples were obtained four hours after the initial clopidogrel loading dose in tubes containing 3,2% sodium citrate. Platelet function assessment was performed using the PFA-200 P2Y system, which simulates the *in vitro* process of primary hemostasis and detects ADP receptor blockade using a membrane coated with ADP (20 μg), PGE-1 (5 ng), and calcium (125 μg). The result is expressed in terms of closure time (CT) measured in seconds, where values <106 s represent HTPR (Siemens Healthcare. 2010. Innovance PFA P2Y.). This value was utilized to define patients into HTPR and non-HTPR.

### Comprehensive custom NGS panel

In this study, we designed a comprehensive custom NGS panel that involved the analysis of 91 SNPs and 28 genes related to clopidogrel metabolism (S1 Table). The SNPs were selected from previous GWAS studies conducted mainly in Europeans significantly associated with adenosine diphosphate-stimulated platelet aggregation [7, 8, 13]. The genes were extracted from the pharmacokinetic and pharmacodynamic pathways described in the PharmGKB database (https://www.pharmgkb.org/).

Agilent NGS custom target enrichment probes were used to design a customized hybrid capture panel to target both SNPs and genes. The design of custom hybridization capture probes for targeted sequencing was conducted using the SureDesign software. To select sequences of interest within an NGS library, we applied hybridization capture-based target enrichment using the SureSelect Custom Tier1 DNA Target Enrichment Probes (Agilent, Santa Clara, CA, USA).

Library preparation was carried out using the SureSelect XT HS2 target enrichment system (Agilent, Santa Clara, CA, USA). For sequencing, the DNA was circularized, and the library underwent denaturation after split oligo ligation. Subsequently, digestion and purification were performed using specific beads. The circularized DNA served as the template for generating DNBs (nanoballs) through the rolling circle amplification process (MGI Tech Co, 2022). DNBs were quantified and then sequenced on the DNBSeqG400 platform. Library preparation and sequencing were conducted by GencellPharma (Bogotá, Colombia).

### Genetic data quality analysis

The sequencing data obtained were analyzed using the hg19 reference genome (GRCh37). We assessed sequencing coverage for a set of 28 genes associated with the drug pathway. For each of these genes, we utilized the ENST codes obtained through Biomart-Ensembl. Exons lacking coding regions were filtered out, and the coordinates of exons to be covered were obtained. To calculate depth and coverage we used the bedcov tool from samtools, using as threshold for coverage 10X, 20X, 50X, 100X, 200X, and 500X. The same procedure was applied to the 91 SNPs with corresponding coordinates, aiming to derive the total read count, which is equivalent to the sum of depths per base. With the goal of achieving a 100% genotyping rate for the SNPs selected in all samples, variant calls with less than 10X were validated through Sanger Sequencing. Primer design was conducted using the Primer-BLAST tool for the amplification via Polymerase Chain Reaction (PCR) of the following SNPs: rs150117487, rs10935838, rs4709967, rs3732759, rs2046934, rs4709968, rs11724226 and rs6831212 (S2 Table). The PCR was standardized, and the amplified products were visualized on 1.2% agarose gels. The amplicons were analyzed through Sanger sequencing, and the presence of the SNP was determined using FinchTV by comparing it to the hg19 reference genome (GRCh37).

### Bioinformatic analysis

Variant Call Format (VCF) files were used for the genotyping of SNPs and for prioritizing variants in the analyzed genes. Variants were prioritized using two filters: a) Clinically validated variants reported in the PharmGKB database (evidence levels 1, 2, and 3) and ClinVar and b) Rare and novel variants (MAF < 2%). For downstream analysis, all variants included in filter a were considered. For filter b, only variants with potential pathogenicity were selected, including Loss-of-Function (LoF) variants (nonsense and frameshift), missense variants, and splicing variants. To predict potential splicing alterations, Adaptive Boosting (ADA) Random Forest (TF) scores (cutoff ≥ 0.6) were used for splice site prediction. To determine the pathogenicity of missense variants, scores from in silico predictors within VarSeq software were considered. These predictors included Sorting Intolerant from Tolerant (SIFT), Polyphen 2, Mutation Taster, Mutation Assessor, and Functional Analysis Through Hidden Markov Models (FATHMM and FATHMM-MKL). Missense variants with 3 out of 6 positive predictors were considered as pathogenic. For the final analysis, variants obtained from filters a and b with a total depth of 8X, allelic representation in heterozygotes of ≥0.25 (minimum 25% of the polymorphic allele), and relevance to clopidogrel response as reported in the PharmGKB database were considered.

### Population genetic analysis and linkage disequilibrium

Allele and genotype frequencies as well as Hardy–Weinberg equilibrium (HWE) were calculated using the SNP-Stats software (https://www.snpstats.net/start.htm) and Haploview 4.2 (https://www.broadinstitute.org/. Deviation from HWE was assessed using a χ2 goodness-of-fit test with 1°of freedom. Allelic frequencies for all molecular variants identified following bioinformatic analysis in the Latin America population were obtained from the genomAD public database version 2.1.1 (https://gnomad.broadinstitute.org/). These frequencies were compared with our data using Chi-square analysis and Yates correction. Statistical significance was determined at p<0,05.

### Statistical analysis

A bivariate analysis was performed to assess the association between clinical and genetic factors and closure time (CT), which was utilized as an indicator of the response to clopidogrel. Patients were categorized into two groups, those with CT<106 seconds were classified as HTPR and those with CT>106 seconds were classified non-HTPR. For categorical variables, Chi-square test was applied, and Odds ratios (OR) and their respective 95% confidence intervals (CI) were calculated. Additionally, a logistic regression model was constructed using the least absolute shrinkage and selection operator (LASSO) regularization method. This model was built using samples from individuals with CT < 106s and individuals with CT > 106s. K-fold cross-validation was employed, dividing the data into three equal-sized folds or subsets. The LASSO model was estimated using the glmnet package. The outcomes of the model were depicted as receiver-operating-characteristic (ROC) curves, with their respective area under the curve (AUC) values. ROC and AUC estimation were performed using the pROC package. Data analysis was conducted using R (v4.1.2). To explore the potential association between SNPs and Mean CT values, comparisons were conducted to assess the potential association between single nucleotide polymorphisms (SNPs) and the numerical variable closure time (CT) using the Mann-Whitney U test with a significance threshold of p < 0,05. For the genetic variants, all statistical analyses were conducted by categorizing genotypes into three groups: a) if they carried 0,1 or 2 polymorphic alleles; b) if they carried 0 or 1 polymorphic allele, and c) if they carried 0 or 2 polymorphic alleles. This context considers an additive model for the association analyses. The co-dominant model (grouping a) proposes that genotypes with 0, 1, or 2 polymorphic alleles are associated with the lowest, intermediate, and highest risk, respectively [14].

### Pharmacogenomic polygenic risk estimation (PgxPRS)

The estimation of PgxPRS was performed using the **β** values obtain from the LASSO analysis. The individual risk score for each patient was determined as described previously and following the standard equation [15].


$$PGxPRS=\overset{N}{\underset{i}{\sum }}\;\beta_{j}*dosage_{ij}$$

N represents the number of SNPs in the score, **β***i* is the effect size (or beta) of variant *i* and *dosage*_*ij*_ is the number of copies of SNP *i* in the genotype of individual **j**.

A linear correlation analysis was conducted between the PgxPRS data and the CT values, establishing the value of r^2^ and the statistical significance of the correlation (Spearman´s rho, p<0,05). The capacity of PGxPRS to differentiate patients HTPR from non-HTPR was evaluated (Mann-Whitney U test, p<0,05).

## Results

### Clinical and demographic characteristics

The study included a total of 70 patients. However, one of the patients was excluded from the final analysis due to the inability to obtain genotyping data, possibly due to low sample quality. Regarding gender distribution, males predominated in the study, accounting for 62.3% of the participants. The most common comorbidities among the patients were hypertension, affecting 63,8% of the participants, followed by dyslipidemia, which affected 56,5% of the patients. Regarding the types of acute coronary syndrome, acute myocardial infarction with ST-segment elevation (STEMI) was the most common, affecting 68,1% of the patients. Furthermore, a significant proportion of the patients exhibited CT<106s categorizing them as HTPR (clopidogrel non-responders), with this category representing 79,7% of the patients in the study. Table 1 summarizes the clinical and sociodemographic characteristics of the study sample.

**Table 1:** Clinical and sociodemographic characteristics.

Characteristic	(n)	%
**Sex**		
Male	43	62,3
Female	26	37,7
**BMI type**		
Normal	27	39,1
Overweight	27	39,1
Obesity	15	21,7
**Diabetes**		
No	46	66,7
Yes	23	33,3
**Hypertension**		
No	25	36,2
Yes	44	63,8
**Dyslipidemia**		
No	29	42
Yes	39	56,5
**Smoking**		
Yes	5	7,2
No	64	92,8
**Alcohol intake**		
No	67	97,1
Yes	2	2,9
**ACS type**		
NSTEMI	47	68,1
STEMI	14	20,3
Unstable angina	8	11,6
**Intervention type**		
PCI	30	43,5
Bypass	18	26,1
Medical management	21	30,4
**Stent**		
No	44	63,8
Yes	25	36,2
**Family history of ACS**		
No	44	63,8
Yes	25	36,2
**Statins**		
No	11	15,9
Yes	58	84,1
**Enoxaparin**		
No	19	27,5
Yes	50	72,5
**ASA**		
No	62	89,9
Yes	7	10,1
***CYP2C19* inducers**		
No	69	100
Yes	0	0
***CYP2C19* inhibitors**		
No	12	17,4
Yes	57	82,6
**PFA interpretation**		
Non-HTPR	14	20,3
HTPR	55	79,7
**Outcome**		
Absent adverse effects	59	85,5
Bleeding	10	14,5
Reinfarction/thrombosis	0	0
**PIatelet count**		
< = 200000	17	24,6
>200000	52	75,4

BMI type: Body Mass Index; ACS type: Acute Coronary Syndrome type; NSTEMI: Non-ST Elevation Myocardial Infarction; STEMI: ST Elevation Myocardial Infarction; PCI: Percutaneous Coronary Intervention; ASA: Acetyl Salicylic Acid; PFA: Platelet Function Analyzer; Non-HTPR: Non-High on Treatment Platelet Reactivity; HTPR: High on Treatment Platelet Reactivity

### Quality analysis of next generation sequencing

For SNPs, the average percentage of samples covered at thresholds of at least 10x, 20x, 50x, 100x, and 200x was consistently above 95,6%, >94,5%, >90,5%, >83.5%, and >78,9%, respectively. Notably, 87% of the analyzed SNPs were genotyped for all patients at a minimum of 10x depth. 96,7% of the SNPs were genotyped for more than 85% samples with a minimum depth of 10x. For the SNPs, rs10935838 and rs150117487, 68,6% and 8,6% patients, respectively, were genotyped at a minimum of 10X (S3 Table). For genes, the average fraction of the target regions covered with at least 10X, 20X, 50X, and 100X was >95%, >92%, >88%, >82%, and >73%, respectively (S4 and S5 Tables). It is important to highlight that for the *CES1* gene, only three exonic regions satisfying the required 10x depth; therefore, this gene was excluded from the study.

### Genomic data obtained from NGS and population genetic analysis

**Fig 1:**
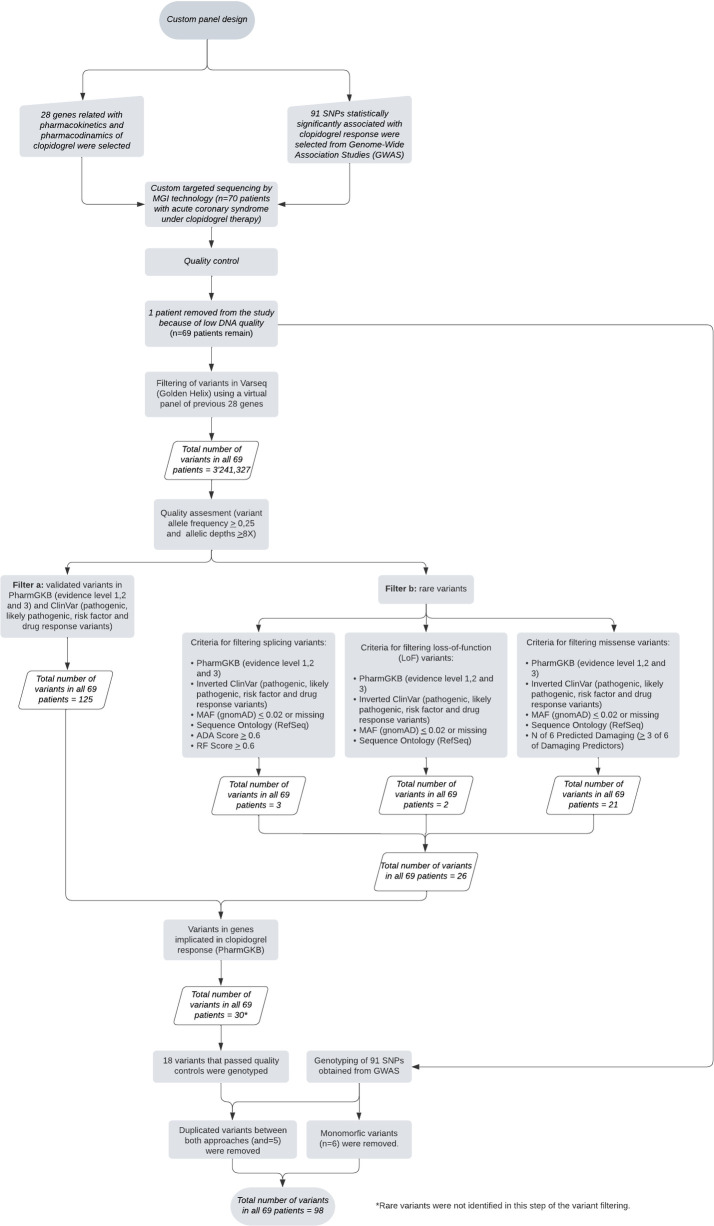
Bioinformatic pipeline.

Of the 91 SNPs included in the custom panel analyzed via NGS, 6 were excluded from the final analysis (rs140410716, rs201441480, rs28365085, rs3749187, rs72558186, and rs74569896) as 100% of the alleles represented in the study population corresponded to wild type. Regarding the analysis of the 28 genes involved in the pharmacokinetics and pharmacodynamics of clopidogrel, a total of 3,241,327 molecular variants were identified in the 69 patients analyzed. After applying the filtering criteria (filter a) described in the methodology, 18 molecular variants were identified (Fig 1). Five of these variants rs2242480, rs41273215, rs57731889, rs6785930 and rs6809699, were common with the SNPs selected from GWAS studies. Following the additional filter (filter b, our analysis has revealed 26 molecular alterations in 14 genes. Among these, 21 (80,7%) are missense, 3 (11,5%) involve splicing, and 2 (7,7%) are insertion-deletion variants. Three of these variants are novel and not cataloged in public databases such as gnomAD v3.1.2 (*ABCB1* NM_000927.5:c.2732C>T, *PTGS1* NM_000962.4:c.94+198_94+338del, *ITGB3* NM_000212.3:c.841C>T) (S6 Table). Given the high sequencing depth achieved through NGS of the customized panel (ranging between 227,9x and 1912,7x), we did not perform Sanger sequencing to confirm any of the rare variants we identified.

A total of 98 variants were included, with 85 of them extracted from previous GWAS studies and 13 obtained from gene analysis after applying bioinformatic filter A as described in the methodology. Population genetic analysis indicated that 96% of the variants are in Hardy-Weinberg equilibrium and exhibited allelic frequencies between 0,007 and 0,9420 (S7 Table). The comparison of the allelic frequencies for the variants in our sample to those reported in the Latin American population in the gnomAD public database indicated that 54 of them (55,1%) presented significant differences (p < 0,05). Interestingly, 63% of these polymorphisms occurred at significantly higher frequencies in the sampled population. Most of them (85,3%) are variants in genes related to the pharmacokinetics and pharmacodynamics of clopidogrel. Notably, rs10306114 in the *PTGS1* had the lowest allele frequency in our population, and rs6809699 in the *P2RY12* gene presented the highest allele frequency, compared with Latin American population allele frequencies, these differences were statistically significant (p<0,05) (S7 Table). Disequilibrium analysis revealed linkage in 14 chromosomes (S1 Fig).

### Clinical-genetic association analysis with HTPR

Analysis of the association between clinical and genetic factors and the response to clopidogrel (established by the closure time-CT) revealed that 5,1% (5/98) of variants were significantly associated (rs1045642, rs2254638, rs2286823, rs2032582 and rs6798347). These SNPs are located within *ABCB1*, *N6AMT1*, *POR*, *ABCB1* and *MED12L* genes respectively. The SNP rs2286823 exhibited the strongest positive association strength (OR: 5,0; 95% CI: 1,02–24,48; p: 0,03) related to carrying 1 or 2 polymorphic alleles (GA+AA). Strong associations were also evidenced for the SNPs rs2032582 (OR: 4,41; 95% CI: 1,20–16,12; p: 0,019) and rs1045642 (OR: 3,38; 95% CI: 0,96–11,9; p: 0,05). A negative association was observed for SNP rs6798347 (OR: 0,15; 95% CI: 0,02–1,08; p: 0,039) related to the genotype carrying 2 polymorphic alleles. Regarding the association of HTPR/Non-HTPR status with non-genetic factors, the only significant association, which was negative, was identified for the stent usage condition (OR: 0,23; 95% CI: 0,07–0,79; p: 0,014). All results are detailed in Table 2.

**Table 2:** Association between genetic factors and clopidogrel response.

Gen	Variable	Genotype	Non-HTPR	HTPR	p.value	OR	IC_lower	IC_Upper
*ABCB1*	rs1045642	AA	6 (42,9%)	10 (18,2%)	0,039	NA	NA	NA
		AG	8 (57,1%)	31 (56,4%)				
		GG	0 (0,0%)	14 (25,5%)				
*ABCB1*	rs1045642_1	AA	6 (42,9%)	10 (18,2%)	0,051	3,38	0,96	11,90
		AG + AA	8 (57,1%)	45 (81,8%)				
*ABCB1*	rs2032582	AA	6 (42,9%)	8 (14,5%)	0,029	NA	NA	NA
		AC	7 (50,0%)	29 (52,7%)				
		CC	1 (7,1%)	18 (32,7%)				
*ABCB1*	rs2032582_1	AA	6 (42,9%)	8 (14,5%)	0,019	4,41	1,20	16,12
		AC + CC	8 (57,1%)	47 (85,5%)				
*N6AMT1*	rs2254638	AA	2 (14,3%)	7 (12,7%)	0,012	NA	NA	NA
		AG	11 (78,6%)	21 (38,2%)				
		GG	1 (7,1%)	27 (49,1%)				
*POR*	rs2286823_1	GG	12 (85,7%)	30 (54,5%)	0,033	5,00	1,02	24,48
		GA + AA	2 (14,3%)	25 (45,5%)				
*MED12L*	rs6798347_3	GG	7 (70%)	31 (93,9%)	0,039	0,15	0,02	1,08
		AA	3 (30%)	2 (6,1%)				
-	Stent	Yes	5 (35,7%)	39 (70,9%)	0,014	0,23	0,07	0,79
		No	9 (64,3%)	16 (29,1%)				

HTPR High on-Treatment Platelet Reactivity. Non-HTPRT Non- High on-Treatment Platelet Reactivity. NA Not AppIicable.

### Genetic association analyses with the mean CT value

We evaluated the association of the 98 prioritized molecular variants with the mean CT values. This approach allowed us to identify 22 SNPs statistically associated (p<0,05). Some of them exhibited a high linkage disequilibrium (genes *NR3C2*, *PDE10A*, *POR* and *ABCB*1) (Table 3 and S1 Fig). It was observed that being a carrier of at least one polymorphic allele resulted in a statistically significant lower CT value compared to the homozygous genotype for the reference allele. Conversely, for SNP rs1330344, non-carriers exhibited decreased CT values (Table 3 and S2 Fig). The polymorphisms were observed at high allelic frequencies in the analyzed population (3,1% to 78,9%), which is relevant given their implication in closure time (CT), an indicator of the response to clopidogrel (S7 Table).

**Table 3:** Association between mean CT values and genetic factors.

Gene	SNP	Genotype	N° of patients	CT mean value
*NR3C2*	rs72729980_3	TT	51	96,84
		CC	3	60,67
	rs907620_3	TT	51	96,84
		CC	3	60,67
*PDE10A*	rs12055399	GG	64	94,89
		GA	5	57,2
	rs4709966	GG	65	94,62
		AA	4	52,25
	rs4709967	TT	64	94,89
		TC	5	57,2
	rs7747284	GG	65	94,62
		GA	4	52,25
	rs9348035	AA	64	94,89
		AG	5	57,2
	rs9348036	GG	64	94,89
		GA	5	57,2
	rs9356381	GG	64	94,89
		GT	5	57,2
	rs9364810	AA	64	94,89
		AG	5	57,2
	rs9365909	AA	64	94,89
		AG	5	57,2
	rs9365910	CC	65	94,62
		CA	4	52,25
*CYP3A5*	rs776746_3	CC	46	88,37
		TT	5	64,6
*POR*	rs2286823_1	GG	42	104
		GA + AA	27	73,74
	rs2302429_3	GG	28	95,82
		AA	3	62,33
*ABCB1*	rs1045642_3	AA	16	107,75
		GG	14	64,07
	rs2032582_3	AA	14	109,29
		CC	15	69,2
*PTGS1*	rs907620_1	CC	5	67,6
		CT + TT	64	94,08
*CYP2C19*	rs12248560_1	CC	56	98,36
		CT + TT	13	65,46
*CDH15*	rs72819363_1	AA	56	96,75
		AC + CC	13	72,38
*CYP4F2*	rs2108622_3	CC	39	95,67
		TT	3	65,67
*N6AMT1*	rs2254638_3	AA	9	100,67
		GG	28	65,89

* Statistical significance. p-value<0,05. NA Not AppIicable.

### Predictive model based on the Least Absolute Shrinkage and Selection Operator algorithm (LASSO)

**Fig 2:**
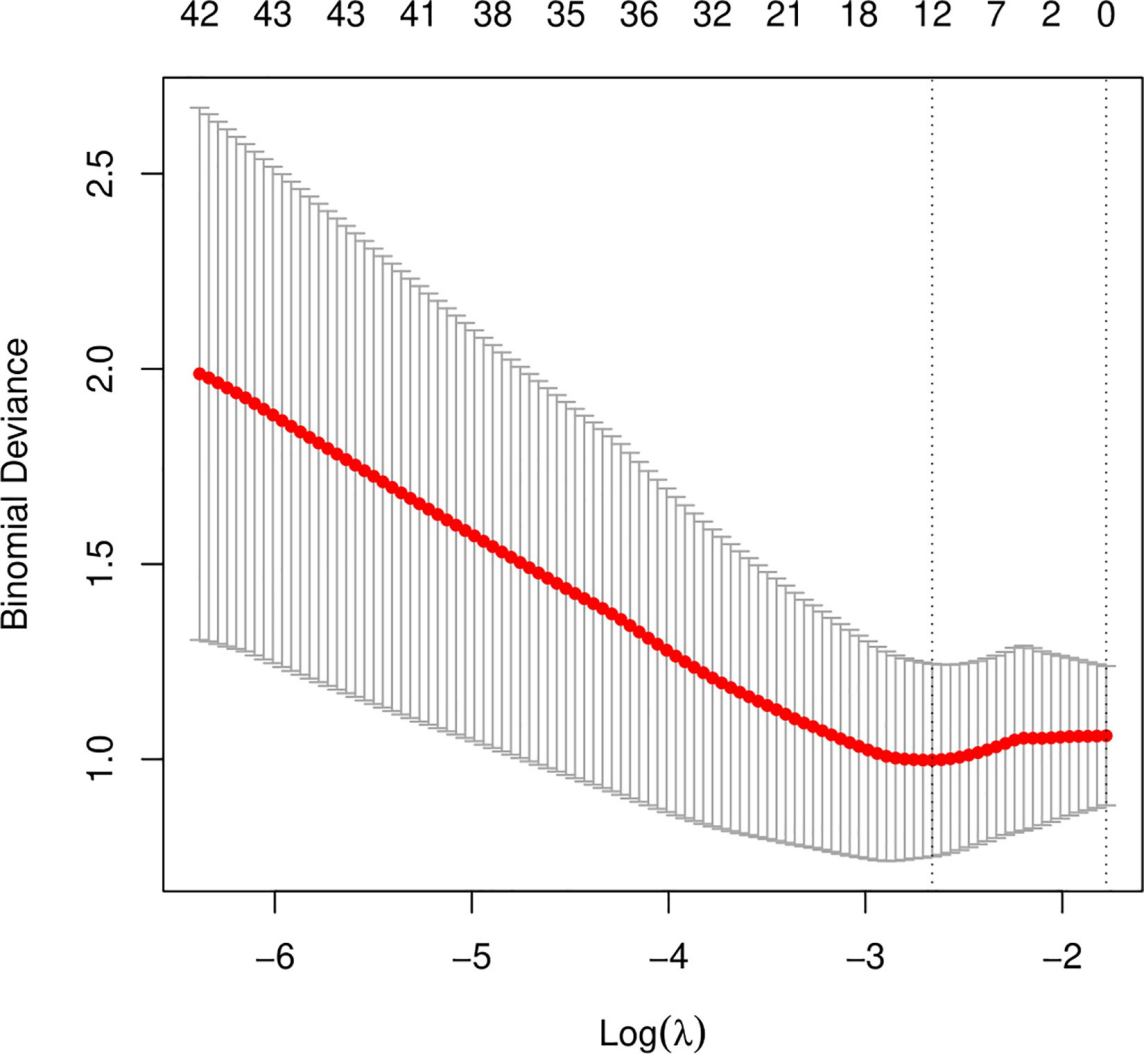
Cross-validation plot for the penalty term λ based on differentially expressed metabolites. Vertical bars represent acceptable maximum and minimum λ values with corresponding mean-squared error and the number of covariates for each model.

**Fig 3:**
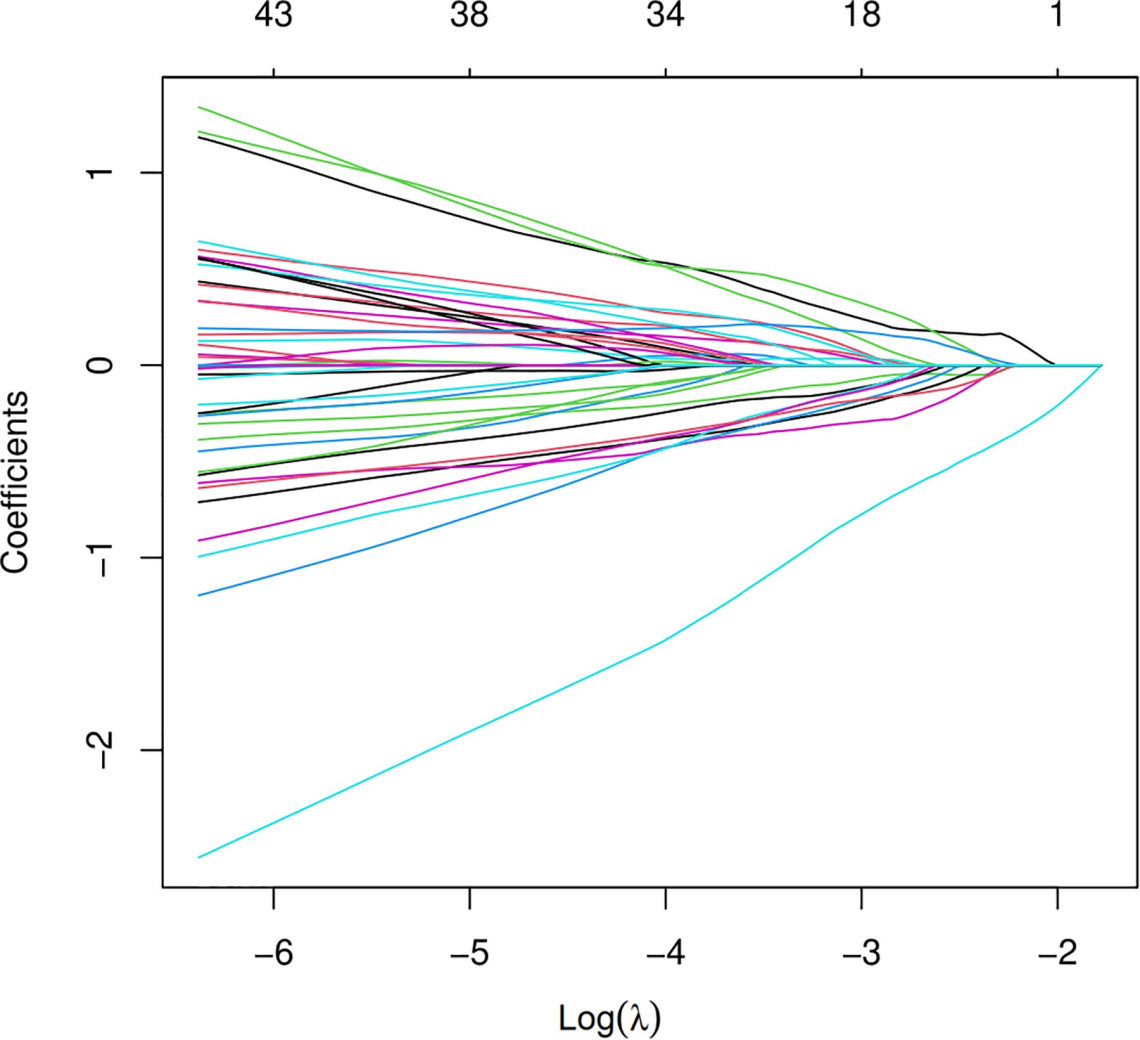
Plots for lasso regression coefficients. Over different values of the penalty parameter λ for each model.

**Fig 4:**
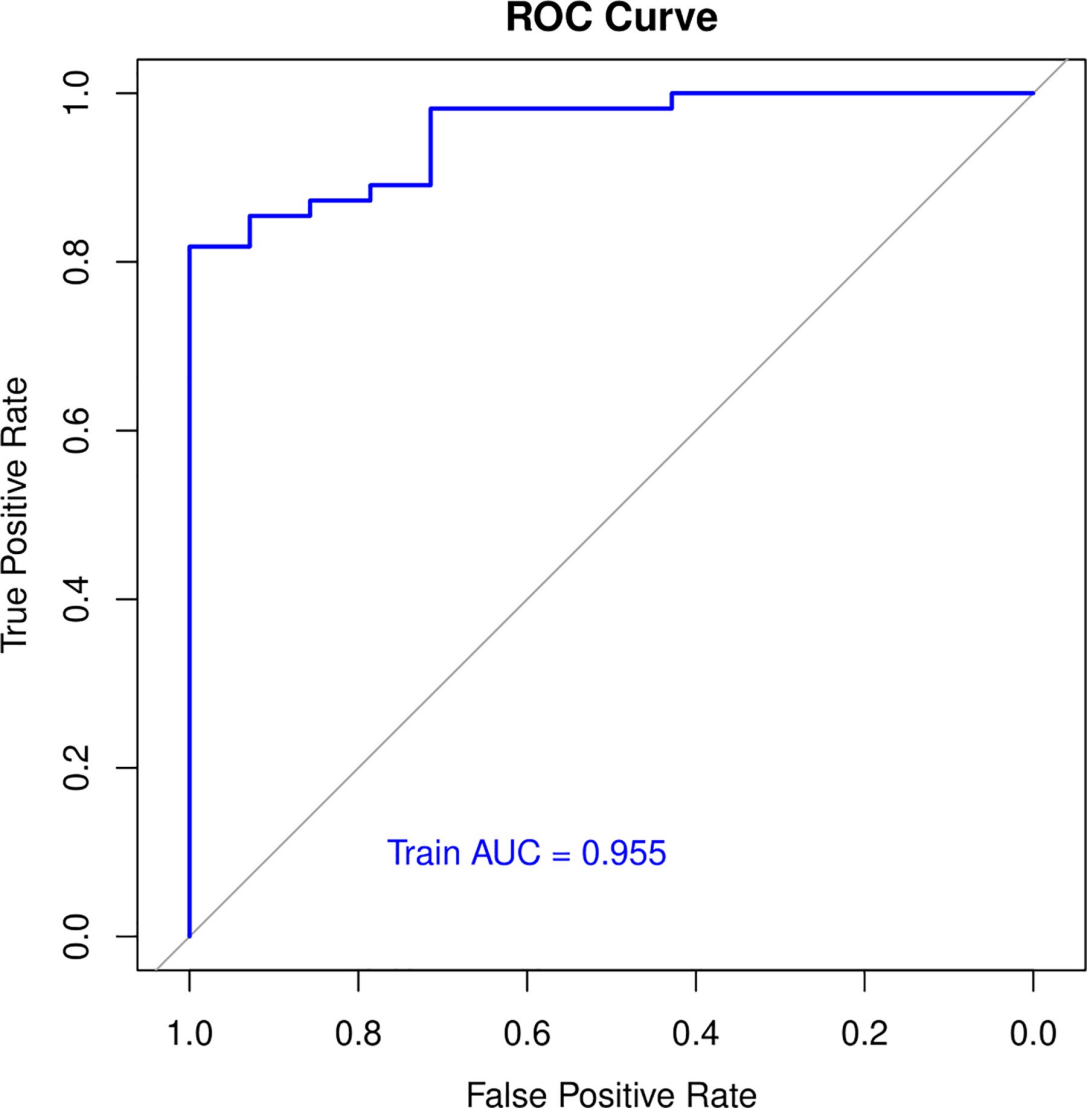
ROC curves for training datasets for the estimated model.

To further construct a predictive model without redundant clinical information and SNPs, we used LASSO regression to narrow down the range of candidate variables. According to mean-square error (Fig 2) and coefficients (Fig 3), we opted for the former λ as it resulted in a better prediction efficiency than the latter λ in the estimated model. The AUC for the model on the training set was 0,955 (Fig 4). The analysis showed that 2 clinical variables (Stent and statins) and 7 SNPs (rs12456693, rs2254638, rs342293, rs57731889, rs6798347, rs6801273 and rs2032582) were the most important for differentiating HTPR from Non-HTPR (Table 4). These SNPs are located within *SLC14A2*, *N6AMT1*, *CTB-30L5*.*1*, *PEAR1*, *MED12L*, *P2RY12* and *ABCB1* respectively.

**Table 4:** Lasso model summary data.

Genotype	Non-HTPR	HTPR	β value
NA	5 (35,7%)	39 (70,9%)	-0,0446
	9 (64,3%)	16 (29,1%)	
NA	0 (0,0%)	11 (20,0%)	-0,0355
	0 (0,0%)	3 (5,5%)	
	14 (100,0%)	41 (74,5%)	
CC	11 (78,6%)	42 (76,4%)	-0,1160
CT	2 (14,3%)	13 (23,6%)	
TT	1 (7,1%)	0 (0,0%)	
AA	2 (14,3%)	7 (12,7%)	0,1787
AG	11 (78,6%)	21 (38,2%)	
GG	1 (7,1%)	27 (49,1%)	
CC	4 (28,6%)	27 (49,1%)	-0,0764
CG	7 (50,0%)	24 (43,6%)	
GG	3 (21,4%)	4 (7,3%)	
CC	9 (64,3%)	34 (61,8%)	-0,0188
CT	3 (21,4%)	19 (34,5%)	
TT	2 (14,3%)	2 (3,6%)	
GG	7 (50,0%)	31 (56,4%)	-0,1389
GA	4 (28,6%)	22 (40,0%)	
AA	3 (21,4%)	2 (3,6%)	
TT	7 (50,0%)	18 (32,7%)	0,1893
TC	3 (21,4%)	29 (52,7%)	
CC	4 (28,6%)	8 (14,5%)	
AA	6 (42,9%)	8 (14,5%)	0,1293
AC + CC	8 (57,1%)	47 (85,5%)	

NA Not AppIicable.

The beta values obtained from LASSO model, representing the coefficients associated with the predictor variables in the linear regression model, demonstrated that three of them (rs2254638, rs6801273 and rs2032582) exhibited positive values, whereas remaining ones were negatives (Table 4). Interestingly, one of them (rs2032582) also displayed a strong association with the HTPR status (OR: 4,41; 95% CI: 1,20–16,12; p: 0,019) as previously described. The SNPs selected by the LASSO model were observed in the study population with high allelic frequencies and two of them (rs342293 and rs6801273) exhibit significantly higher frequencies in our population compared to those in Latin American populations described in the gnomAD database (S7 Table). None of them exhibit linkage disequilibrium.

### Pharmacogenomics polygenic risk score (PgxPRS)

We developed a PgXPRS model considering individual effect sizes (β coefficients) and genotypes of the SNPs identified as predictor variables in the linear regression model (LASSO). The beta coefficients of each SNP used in PGxPRS determination are described in Table 4. Individual scores were determined for the 69 patients included in the study, with values ranging from -0,21119257 to 0,78887658 (S8 Table).

**Fig 5:**
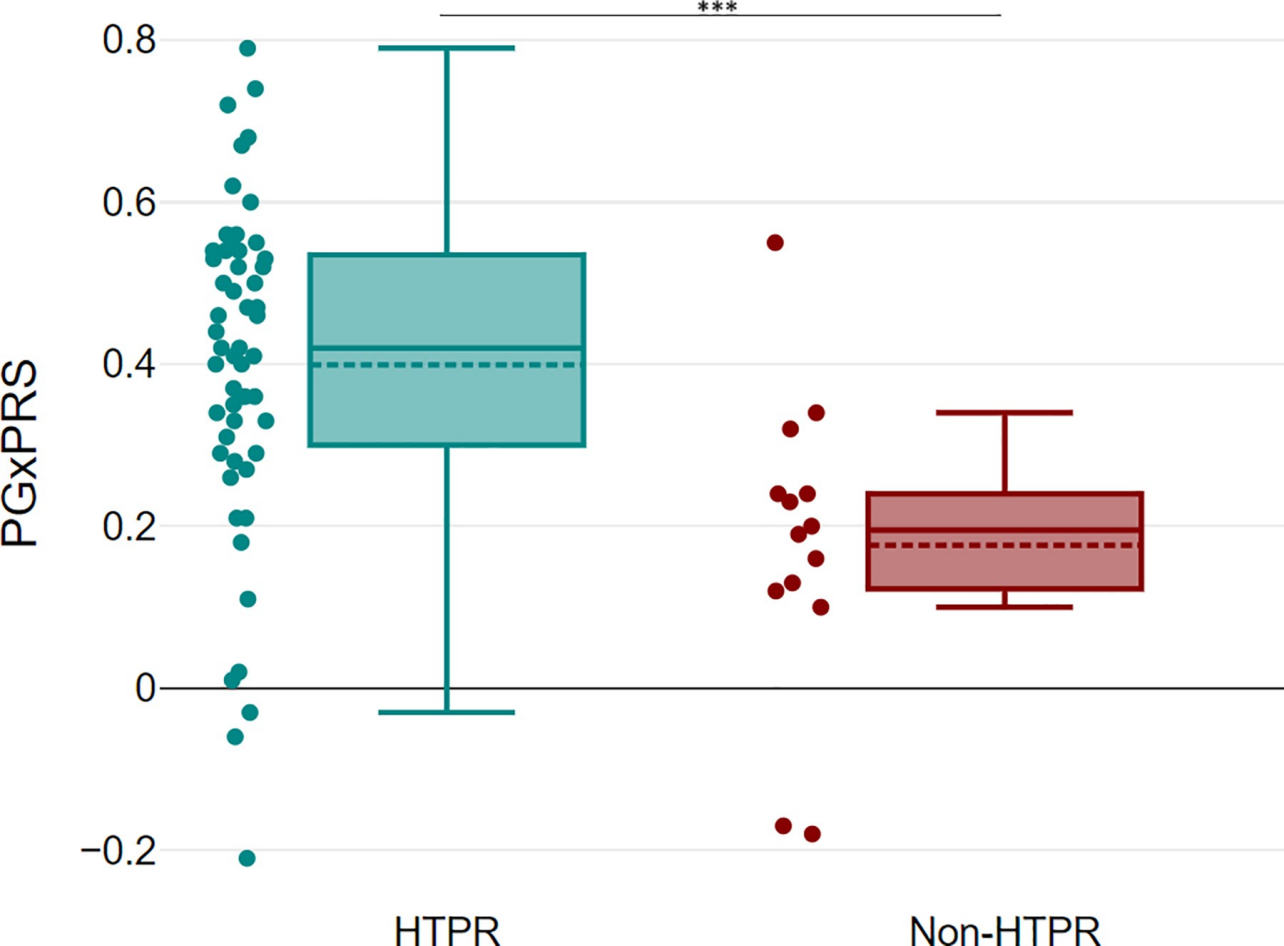
Boxplot PGxPRS vs HTPR condition. Boxplot illustrating the relationship between responders and non-responders with respect to PGxPRS. The Mann-Whitney test reveals a statistically significant difference (p<0.0003).

**Fig 6:**
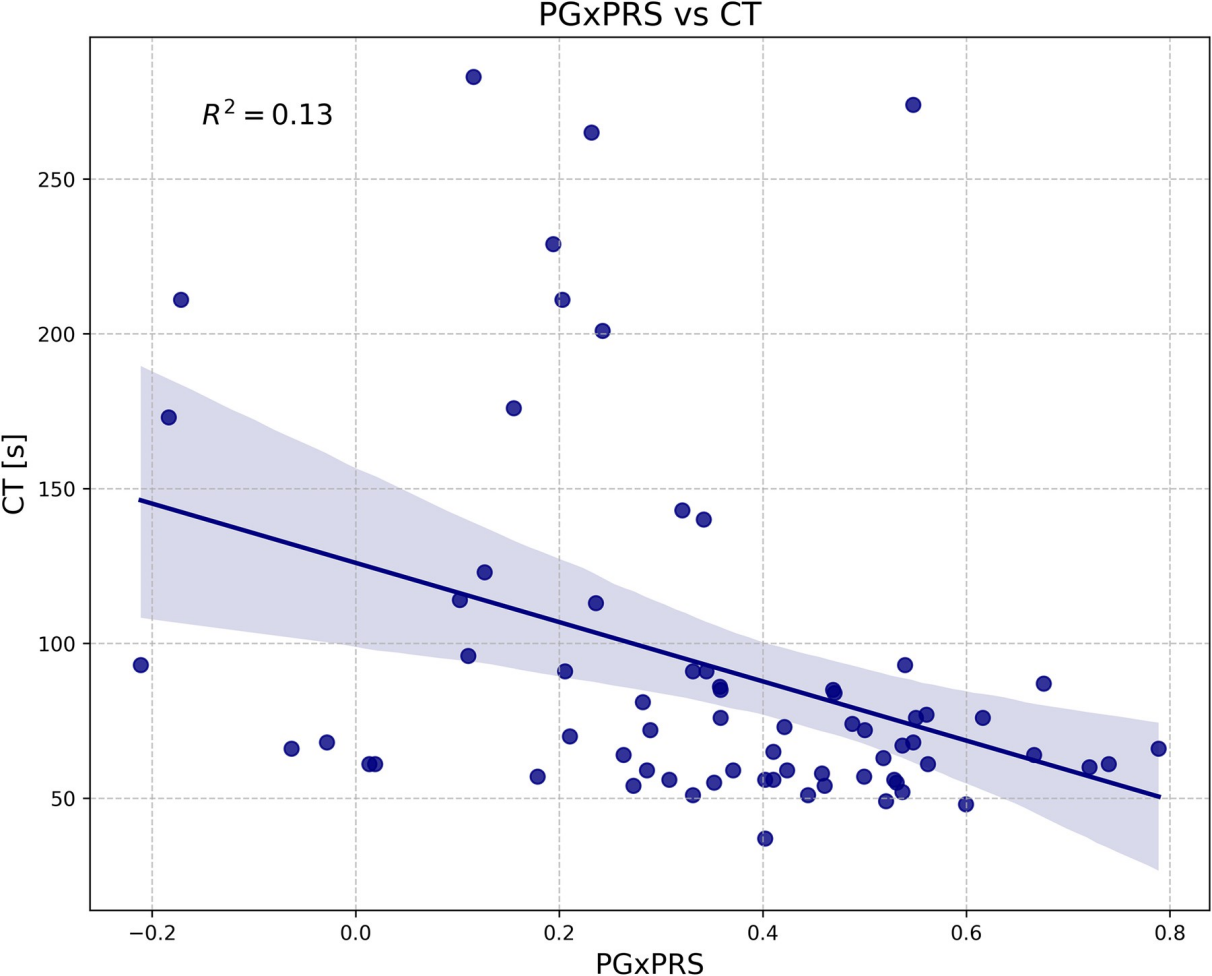
Scatterplot between polygenic risk score (PGxPRS) and obturation time (CT) with the added regression line illustrating the Spearman’s rho correlation (p<0.05).

The comparison of PGxPRS values between HTPR and non-HTPR patients demonstrated a statistically significant difference, thus highlighting the model’s ability for distinguishing these two groups (p-value: 0,0002962) (Fig 5). Finally, a negative correlation was observed between PGxPRS and CT (p<0,001). The coefficient of determination (R^2^) was 0,13, indicating that approximately 13% of the variability in CT values can be attributed to variability in PgxPRS (Fig 6).

## Discussion

Clopidogrel continues to be the most extensively used antiplatelet drug globally [3], however, there is documented high variability in drug response, with an estimated 70% of this variability attributed to genetic factors [4]. It has been established that CYP2C19*2, a well-studied loss-of-function allele, account for only 12% of the variability in Europeans, implying that unexplored genetic variant impact clopidogrel response [16–19]. Based on findings of SNPs associated with clopidogrel response evaluated through GWAS [5, 7, 8, 13] and the analysis of the entire coding region and adjacent introns of drug metabolism-related genes (https://www.pharmgkb.org/), we designed a comprehensive pharmacogenetic panel (PGx) analyzed through NGS. To our knowledge, this approach has not been previously explored. The customized panel offers great flexibility in analysis, allowing the study of predefined gene sets and SNPs for specific phenotypes, with significant advantages such as the ability to simultaneously sequence multiple loci with great depth of coverage [20]. These findings support the use of current molecular methodologies based on targeted sequencing, which provide the potential to identify common, novel, and rare variants in pharmacogenetic interest genes [21].

Our designed PGx panel enabled the genotyping of 91 SNPs previously suggested to be associated with clopidogrel response by GWAS, six of them were monomorphic and therefore unsuitable for analysis. It is not surprising to identify these differences when considering that GWAS studies involve different populations than the underrepresented Latin American population [8, 13]. It is of interest that genetic architecture differences among populations determine that some markers are non-polymorphic or rare, and thus are related to the observed lack of replication in transethnic studies involving non-European ancestral populations [22].

Statistically significant differences were evident among the allele frequencies of 55,1% of the analyzed SNPs in our population (ranging from 0,7% to 94,2%) compared to those reported in the Latin American population in the gnomAD database, 63% of them appeared at higher frequencies (p<0,05) (S7 Table). Notably, we have identified these population differences in other studies conducted with samples from Colombian population, where genes related to the absorption, distribution, metabolism, and excretion (ADME) of drugs, response to anesthetics, and the coding region of *CYP2C19* have been evaluated [11, 12, 23]. In all these studies, allele frequency comparisons have been made with the gnomAD database, Latino/Admixed American, which has determined that the described population consists of individuals with a wide range of admixture and significant variations in frequencies compared to other populations (https://gnomad.broadinstitute.org/). The population differences found in this, and other studies may result from the lack of representation of all Hispanic groups in this database, highlighting the current need for genetic analysis in our Latin American populations.

We conducted analyses to determine the impact of genetic variables on clopidogrel responsiveness, utilizing the CT value, that has been described as a potential predictive biomarker [24–26]. Bivariate analyses indicated that five SNPs were significantly associated. The SNPs rs2286823 (*POR-*c.1239+20G>A), rs2032582 (*ABCB1*-c.2677G>T), and rs1045642 (*ABCB1*-c.3435C>T) exhibited the strongest positive association (Table 2). Previous studies have shown that the *POR* gene plays a crucial role in interindividual differences in drug metabolism by affecting the activity of CYP enzymes [27]. Analyses in knockout mouse models have demonstrated that the loss of POR significantly reduces the metabolic capability of CYP enzymes, including CYP2C19, responsible for approximately 45% of clopidogrel conversion to its active metabolite [28]. In this context, the SNP *POR*- rs2286823, associated with decreased protein expression, can potentially diminish catalysis by CYP2C19 to barely detectable levels, like the effect caused by variants related to lower POR activity, such as A287P and R457H [29]. Our results are consistent with Shiraska et al., 2016, who proposed the significance of *POR* variants in individualizing clopidogrel therapy. In our population, carrying polymorphic alleles is associated with a fourfold risk of developing HTPR [30].

Regarding the implication of *ABCB1* gene variants rs2032582 and rs1045642 in the response to clopidogrel, our findings are in concordance with several literature reports indicating their association with a poor response to clopidogrel. Our results indicate that carriers of polymorphic alleles for *ABCB1* c.2677G>T or *ABCB1* c.3435C>T confer a 3,4- and 2,3-times higher risk of an inadequate response to clopidogrel (HTPR). For carriers of the *ABCB1*- 3435T variant, lower peak plasma concentration (Cmax) and total area under the plasma concentration-time curve (AUC) values of clopidogrel and its active metabolite were determined compared to non-carriers, suggesting that the *ABCB1* C3435T genotype influences clopidogrel absorption and active metabolite formation [31–33]. Similarly, carriers of *ABCB1* rs2032582 were also correlated with a decreased response to clopidogrel due to higher-than-expected platelet function compared to non-carriers [10]. In summary, this evidence supports our findings and demonstrates the functional impact of these variants on platelet reactivity, which was noteworthy in our population.

In addition, less-studied variants have demonstrated significant implications for HTPR, one of these corresponds to the SNP rs2254638 in the *N6AMT1* gene, for which a high proportion of homozygotes for the polymorphic allele (49,1%) was associated with a poor antiplatelet response (Table 2). *N6AMT1* encodes for N (6)-adenine-specific DNA methyltransferase involved in the methylation of the release factor I [34] and has been previously linked to increased clopidogrel resistance [35].

Our analysis revealed that rs6798347 (*P2RY12* c.−281−3614C>T) constitutes a protective allele (OR: 0,15; 95% CI: 0,02–1,08; p: 0,039), in accordance with previous reports. This SNP, located in the promotor region of the gene, has been associated with a significantly lower incidence of HTPR compared to non-carriers [36, 37].

Given the potential association between reduced CT and a poorer prognosis in terms of clopidogrel response, we decided to investigate the relationship of genetic polymorphism with the mean CT value. This approach enabled us to identify statistically significant differences with 22 SNPs (Table 3). Some of the SNPs that exhibited a positive association (p<0,05) were also identified in the previously discussed analysis (HTPR vs. non-HTPR condition) (*N6AMT1*: rs2254638; *POR*: rs2286823; *ABCB1*: rs1045642, rs2032582), supporting their significant influence on clopidogrel responsiveness.

An innovative discovery is the identification of several SNPs in the *PDE10A* gene to be associated with lower CT values (p: 0,0012), which, to our knowledge, had not been previously described in the literature. PDE10A is a member of the PDE10 protein family that catalyzes the hydrolysis of cAMP and cGMP. While the literature has focused on its role in psychiatric and neurological disorders, its involvement in smooth muscle cell (SMC)-like cell proliferation, injury-induced intimal thickening, and accelerated atherosclerosis has been recently documented [38]. It is estimated that the regulation of *PDE10A* gene expression is influenced by highly conserved putative intronic transcriptional sequences (https://grantome.com/grant/NIH/R01-HL134910-01). Therefore, the molecular variations identified in our patients could be related to this up-regulation. In terms of clopidogrel response, it is plausible to hypothesize that intimal hyperplasia may affect treatment efficacy due to its association with a higher load of activated platelets and, consequently, increased resistance to platelet inhibition, suggesting that patients with *PDE10A* SNPs may require adjustments in antiplatelet therapy. Notably, the molecular variants of the PDE10A gene associated with the CT mean value are in LD (Table 3 and S1 Fig). This finding enables the translational application of our results by allowing the selection of a single nucleotide polymorphism (SNP) that optimizes the information retained in a genomic region while reducing the genotyping effort and simplifying the analysis. It has been estimated that with sample sizes ranging from 50 to 100 individuals, consistent results can be achieved to identify these LD patterns. This enables genotyping fewer markers that yield redundant information due to extensive LD between nearby SNPs [39].

Other SNPs related to lower CT values have previously been linked to clopidogrel response, such as the case of *CYP3A5*-rs776746, and carriers of the polymorphic allele are estimated to have a high hepatic content of CYP3A, potentially affecting the drug [40]. We observed that rs2108622 in the *CYP4F2* gene showed lower CT values in TT homozygous patients, an allele associated with loss of function. This is explained by the interaction of a metabolite resulting from CYP4F2 monooxygenase-mediated W-hydroxylation of arachidonic acid with TxA2-induced platelet aggregation, thereby reducing platelet activation and aggregation through the platelet TxA2 receptor [41]. It is noteworthy that these genes, *CYP3A5* and *CYP4F2*, play a significant role in the response to tacrolimus and warfarin, respectively. However, our study, along with other literature reports, highlight their involvement in the response to clopidogrel. This emphasizes the importance of considering these genes in the monitoring of this antiplatelet agent.

For findings derived from case-control association analyses, it is essential to consider the influence of population stratification due to undetected population structure differences in ancestry that may lead to spurious associations [42]. Although our study did not involve ancestry informative markers (AIMs) analysis, we estimate that our patients exhibit a similar genetic background without the influence of factors such as genetic isolation or non-random mating, which are determinants of population stratification. The sample analyzed originates from the Andean region, where high inter-individual variation typical of other regions in the country has not been identified [43]. Additionally, it has been suggested that approaches such as family-based designs or genomic control are necessary when the identified genetic effect is very small (OR < 1,20) [42]. However, our findings demonstrated much higher odds ratios (ORs) ranging from 3.8 to 5.0 (Table 2).

Despite the benefits derived from bivariate analyses, the relevance of analyzing models that include all genetic and non-genetic variables simultaneously has been emphasized. With this perspective, we conducted a combined analysis involving 30 clinical variables and 85 SNPs to explore a predictive model for HTPR and non-HTPR patients. This combined effect was evaluated using the machine learning approach, The Least Absolute Shrinkage and Selection Operator penalized regression model (LASSO).

LASSO regression is commonly employed to reduce model complexity by applying a penalty factor to emphasize more important variables. This model can reduce the effect of variables to zero, thereby decreasing the number of variables in a model [44]. LASSO has the advantage of avoiding overfitting and selecting only one feature from a group of correlated features [45].

This supervised machine learning approach is a regression method that has been used in pharmacogenomic prediction models for Warfarin dosage, demonstrating superior performance compared to algorithms based on multivariate linear regression. These findings, along with those of other authors, demonstrate that in pharmacogenetics, machine learning approaches show satisfactory performance in predicting drug responses in various fields such as cancer, depression, and anticoagulant therapy [45–47]. Results derived from the AUC value (Fig 4) demonstrate that our model exhibits a high predictive capability (0,955) in distinguishing between HTPR and non-HTPR patients. This elevated AUC value, indicating that the LASSO model effectively separates phenotypic classes even with penalization and variable selection, suggests that the two non-genetic characteristics (stent and statins) and the 7 SNPs (rs12456693, rs2254638, rs342293, rs57731889, rs6798347, rs6801273, and rs2032582) selected by LASSO are relevant for the classification of HTPR and non-HTPR (Table 4).

The data derived from the LASSO model enabled the generation of a PgxPRS related to the HTPR or non-HTPR condition. While several literature reports use effect size estimation derived from GWAS data, our estimation from the LASSO model is innovative and has recently been successfully applied in generating risk scores for patient stratification in susceptibility to ovarian cancer and Alzheimer’s disease [48, 49]. While drug dosing guidelines have been based on the analysis of one or a few SNPs, polygenic risk scores have emerged as a promising tool given the complex interaction and polygenic nature of drug response [9, 50, 51]. The PgxPRS developed by us enabled us to establish that HTPR patients with low CT values (Figs 5 and 6) exhibited the highest risk score values (Fig 5) (p<0,05). Given the relationship between CT value and inadequate clopidogrel response, our model represents a potential tool in clinical practice for patient risk stratification.

Significantly, our genomic approach using a custom panel evaluated through NGS has not been addressed in the mentioned studies or others in the literature. In our population, SNPs not described in previous studies developing PgxPRS were identified (rs12456693, rs2254638, rs342293, rs57731889, rs6798347, rs6801273). The majority of PGxPRS have involved predominantly European populations, so extrapolation to populations of different ethnic origins should be done with caution [8].

Surprisingly, for our population, the impact of CYP2C19*2 was not evident in any of the analyses derived from the study. This finding can be primarily explained by the allele frequency in the study population (7,9%), which is significantly lower compared to other populations such as European (14.6%), Asian (30%), or African American (17,5%) populations [52]. This result is consistent with other study conducted by us in larger cohorts of individuals from the Colombian population [11]. The impact of SNPs may be influenced by their frequency across populations, emphasizing the need to incorporate comprehensive genomic studies involving a larger number of genetic markers potentially related to clopidogrel response.

Regarding the analysis of genes related to clopidogrel metabolism, the prioritization of molecular variants established by the Filter A identified 13 pharmacogenetically actionable polymorphisms in response to clopidogrel, as reported by the ClinVar and PharmKGB databases (evidence levels 1, 2, and 3). These variants were presented with allele frequencies ranging from 2,1% to 47,8%, and 76,9% of them showed statistically significant differences compared to those reported for the Latin American population in the gnomAD database, highlighting the importance of conducting specific population characterization and contributing to fill this knowledge gap in our population. These variants were predominantly represented by missense variants (80,7%), in accordance with previous studies, where an enrichment of pharmacogenetic variation by rare missense variants has been observed. This enrichment is primarily explained by low evolutionary constraint assumed for this type of genomic variation [53]. A previously reported study by our group characterizing ADME (absorption, distribution, metabolism, and excretion) genes through NGS revealed that rare missense variants are 2,1 times more frequent than common ones in our population. This emphasizes that such variants serve as a significant source of variability in pharmacogenes and likely account for unexplained interindividual variability in drug response [11]. The impact of these variants should be analyzed in larger patient cohorts or through functional validation, and their clinical application should be considered carefully.

Our work presents the first study using a custom panel of SNPs and genes related to clopidogrel response to develop models that establish associations and generate PgxPRS with molecular variants related to CT, a biomarker for clopidogrel response. This exploratory study proposes a PgxPRS that, when validated, becomes an invaluable tool in translational medicine applicable to stratifying patients at risk of not responding appropriately to antiplatelet treatment.

### Study limitations

The present study has several noteworthy limitations. Firstly, the limited sample size did not allow for a division to achieve training and validation through the LASSO model. For this reason, our results, demonstrating a high discriminatory power between HTPR and non-HTPR patients, should be considered exploratory. Secondly, our sample size may affect the accuracy of PgxPRS prediction, as a large discovery sample is required to determine the expected contribution of each SNP to the polygenic score for clopidogrel response. Thirdly, no replication data were included, so the conclusions derived from our study, which is the first to use a customized panel approach and LASSO analysis, should be interpreted with caution. The functional impact of rare variants was determined using an I*n-Silico* approach, therefore, functional validation or analysis in large patient cohorts is necessary. Finally, our results are derived from clopidogrel response determined through platelet reactivity analysis by assessing CT, so follow-up for the verification of major adverse cardiac events may be of great clinical utility.

## Conclusions

We have conducted the first pharmacogenomic study based on the analysis through NGS of multiple SNPs and genes related to platelet reactivity in patients treated with clopidogrel. Through case-control association analysis, we determined statistically significant associations within our population with SNPs rs1045642, rs2032582, and rs2286823. Similarly, molecular variants of the genes *NR3C2*, *PDE10A*, *CYP3A5*, *POR*, *ABCB1*, *PTGS1*, *CYP2C29*, *CDH15*, *CYP4F2*, and *N6AMT1* show association with CT values, a “surrogate” biomarker of clopidogrel response. The generation of a PgxPRS for the discrimination of HTPR and non-HTPR support the polygenic implication of the response to clopidogrel. Enhancing our understanding of the genetic architecture influencing variability in response to antiplatelet pharmacotherapy is crucial for addressing the challenge of inadequate responses to clopidogrel and exploring alternative therapeutic options. The exploration of such alternatives needs advancements in research. This pursuit aims not only to optimize current antiplatelet strategies, but also to pave the way for personalized medicine, where treatment decisions can be tailored based on an individual’s genetic profile.

## Supporting information

S1 Fig Linkage disequilibrium analysis. (PDF)

S2 Fig Boxplots mean CT values related to genotype of SNPs. (PDF)

S1 Table Comprehensive custom next generation sequencing panel. (XLSX)

S2 Table Primers sequences used in sanger analysis. (XLSX)

S3 Table Bioinformatic quality control for SNPs. (XLSX)

S4 Table Sequencing quality for genes of interest per patient. (XLSX)

S5 Table Sequencing quality for genes of interest. (XLSX)

S6 Table Rare variants obtained through NGS. (XLSX)

S7 Table Population genetic analysis. (XLSX)

S8 Table PGxPRS values per patient. (XLSX)
