# Supplementary figures and images for: The polygenic implication of clopidogrel responsiveness: Insights from platelet reactivity analysis and next-generation sequencing
Source: PLoS One. 2024 Jul 11;19(7):e0306445. doi: 10.1371/journal.pone.0306445 (PMC11239111; doi:10.1371/journal.pone.0306445)

**Figure S1.** Linkage disequilibrium analysis through Haploview 4.2.

**Chr4**

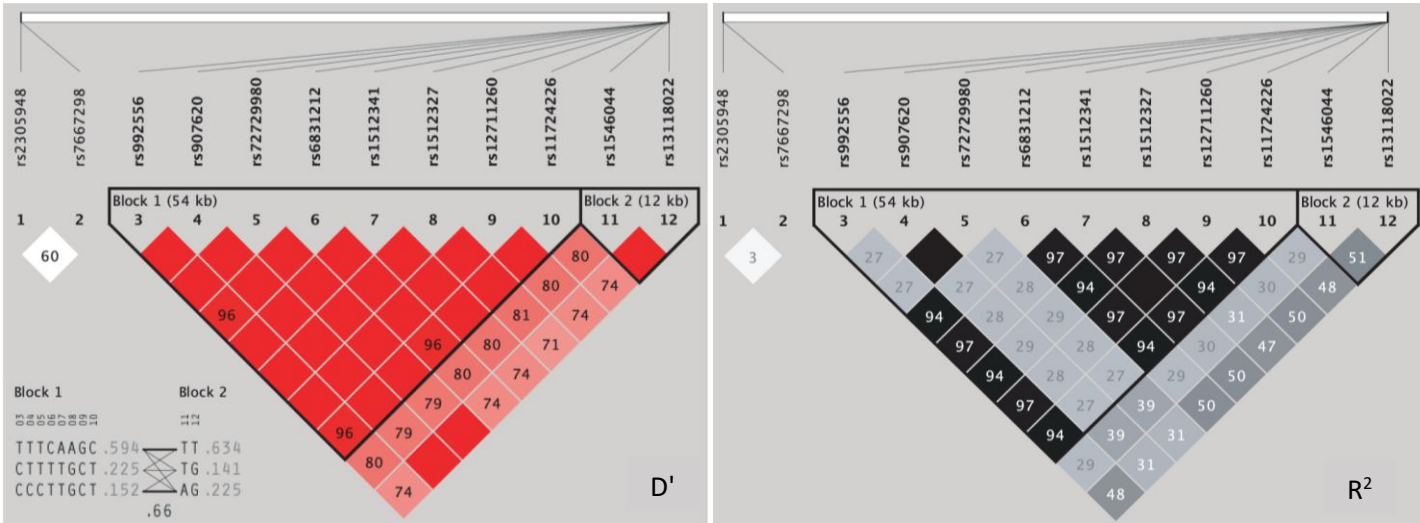

**Chr6**

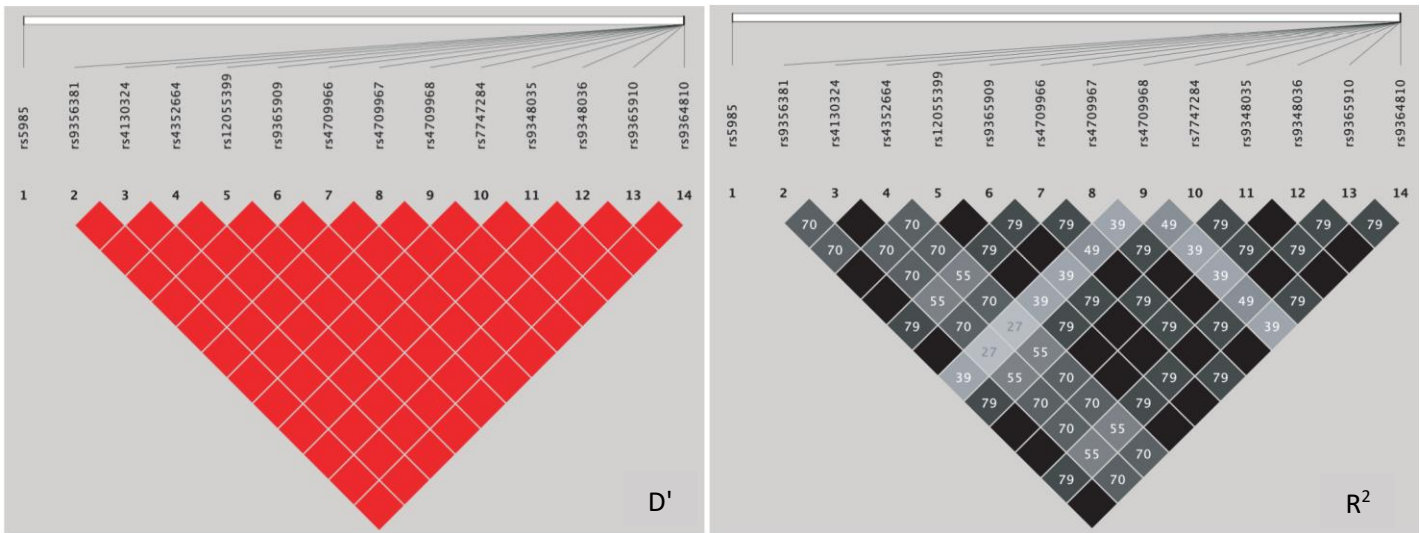

**Chr7**

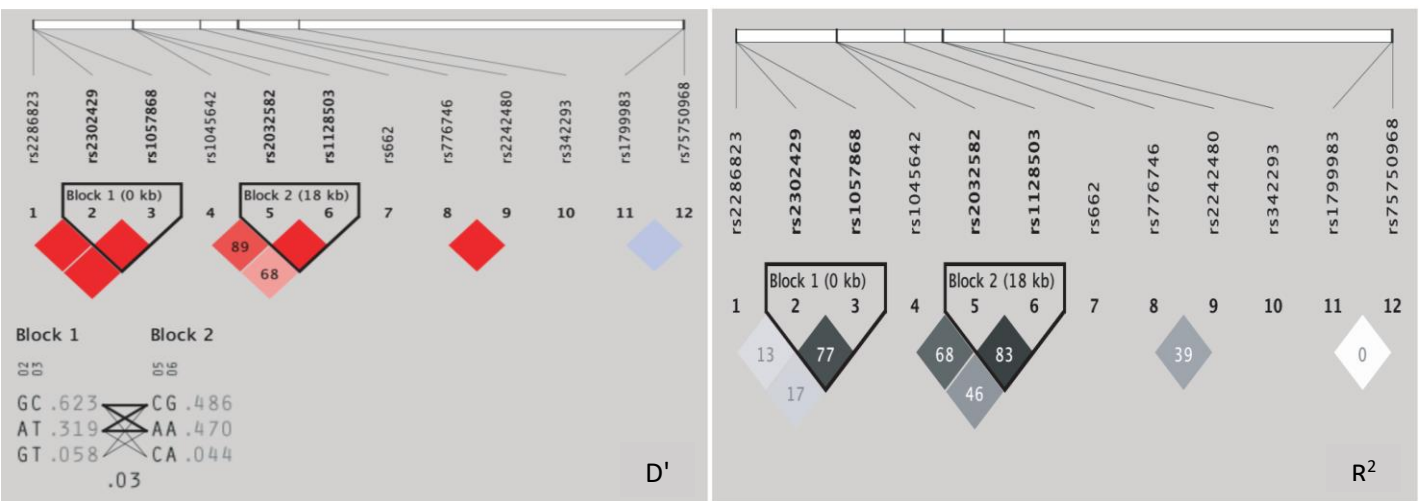

Chr9

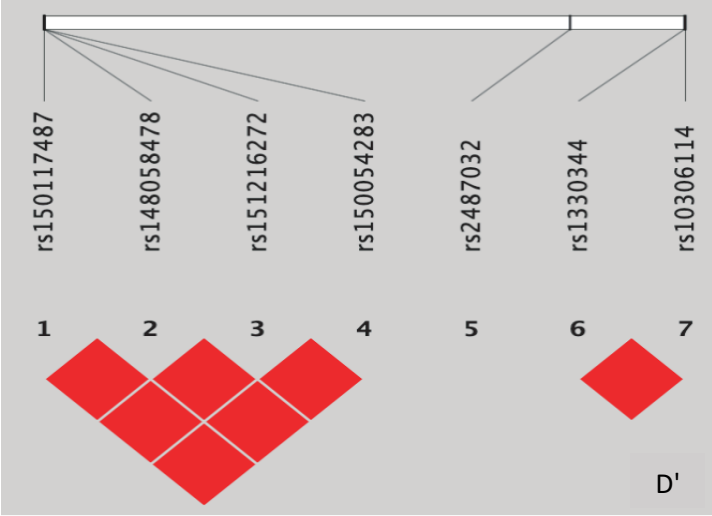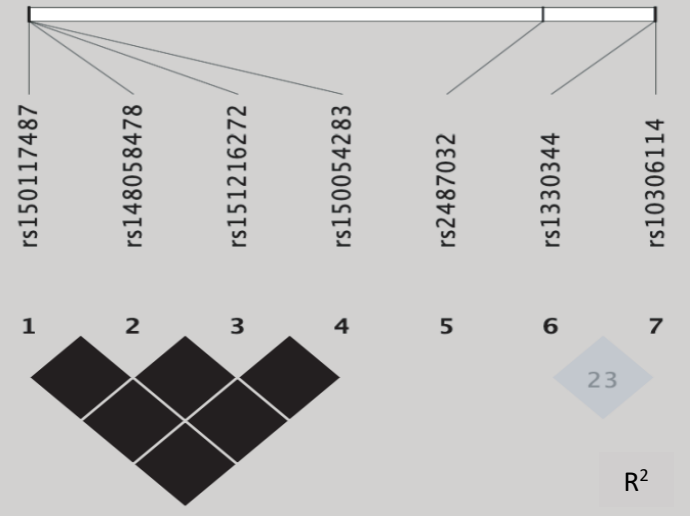

Chr10

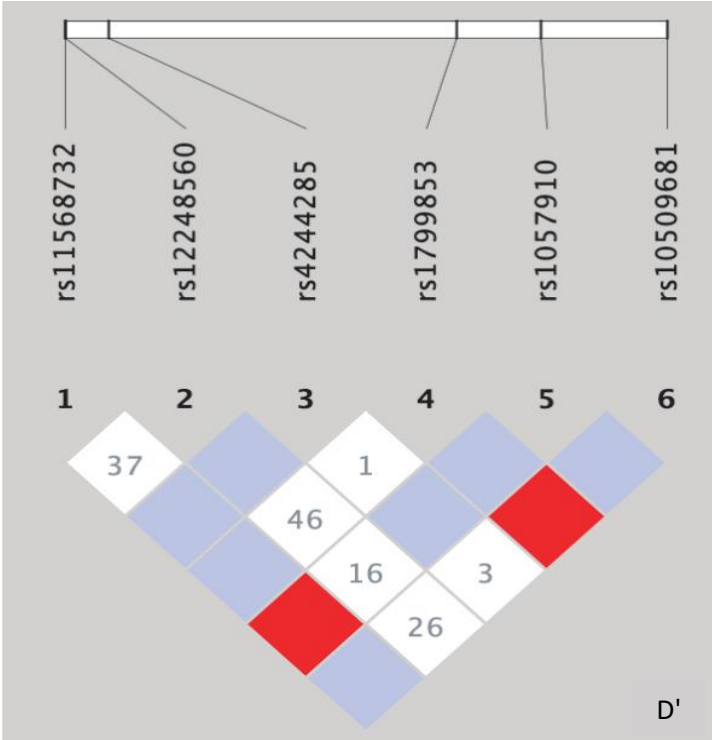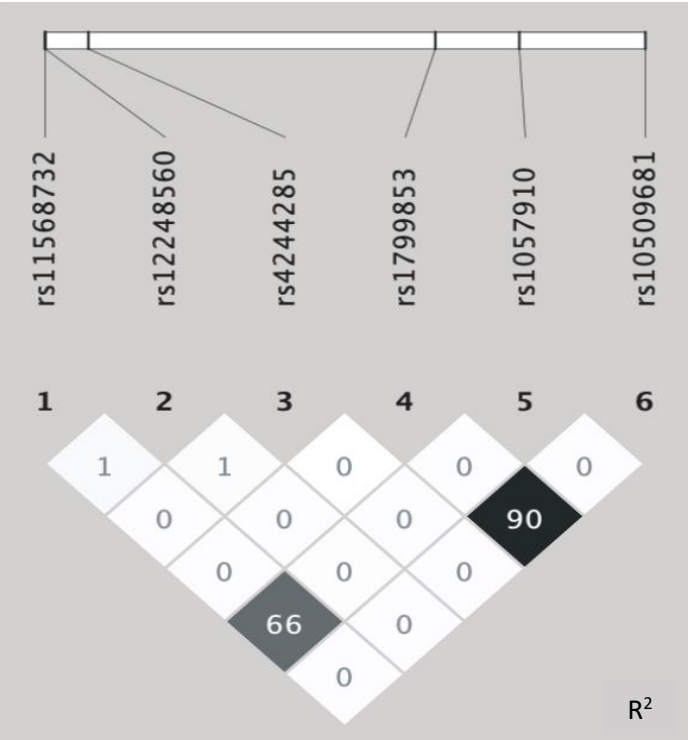

Chr16

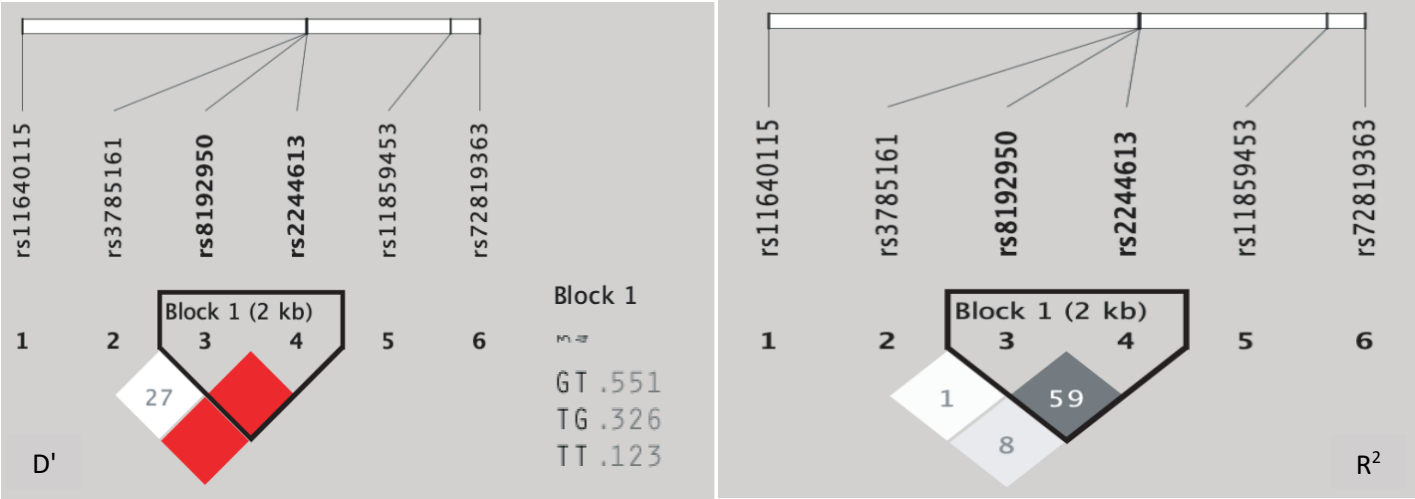

Chr19

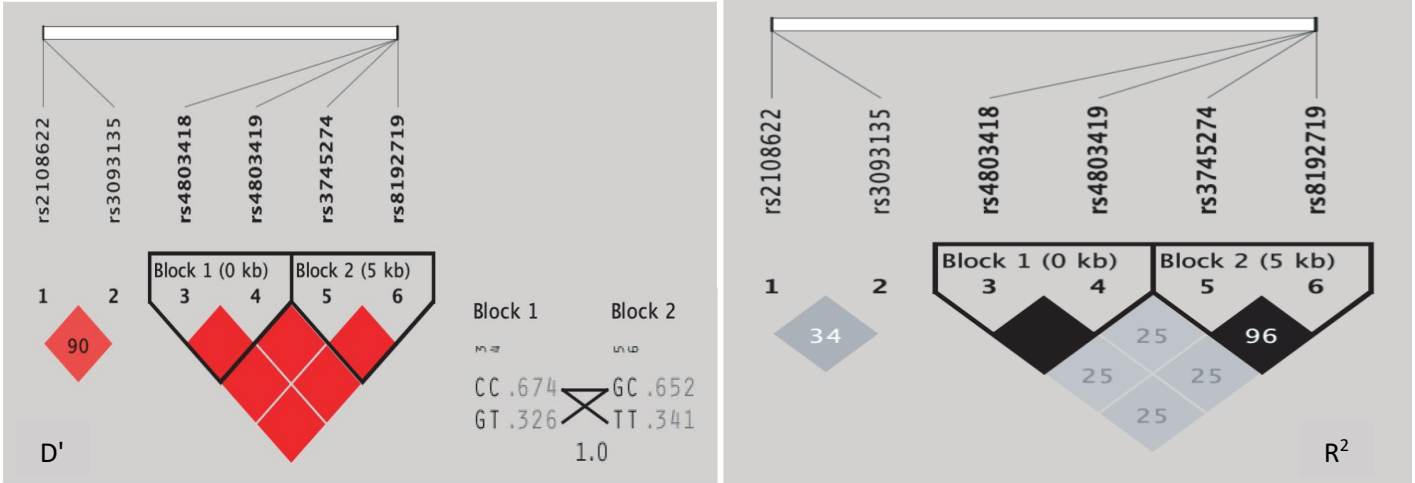

Chr1

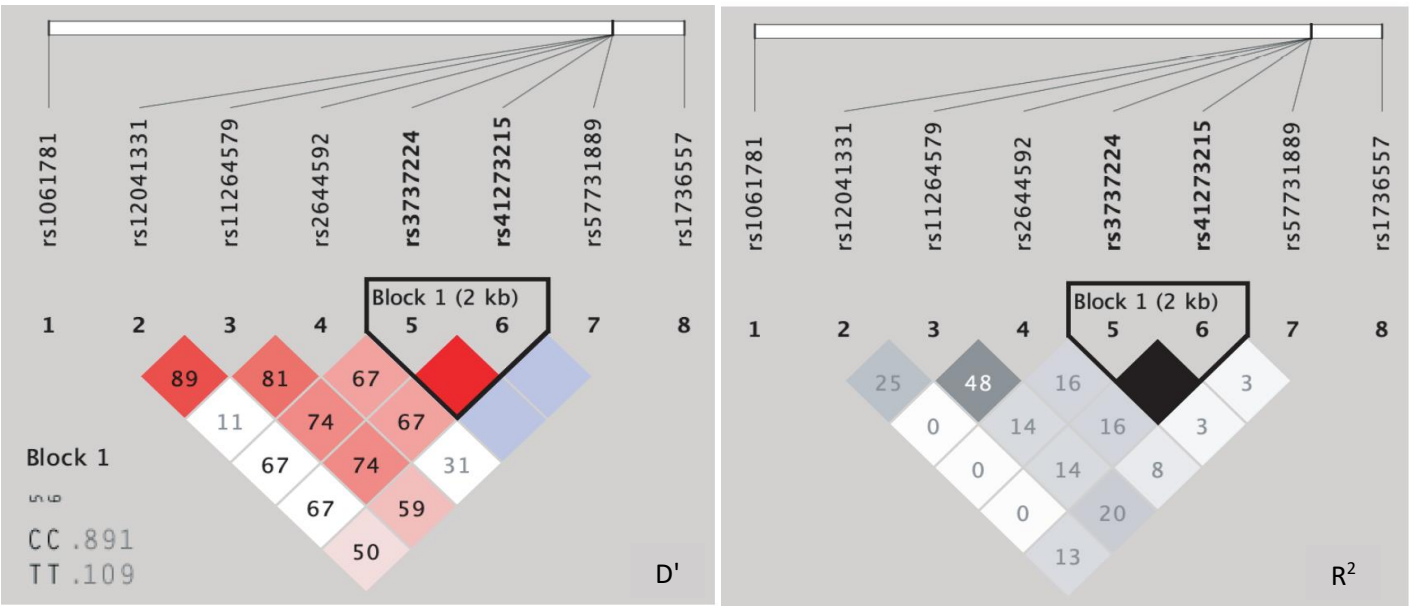

Chr3

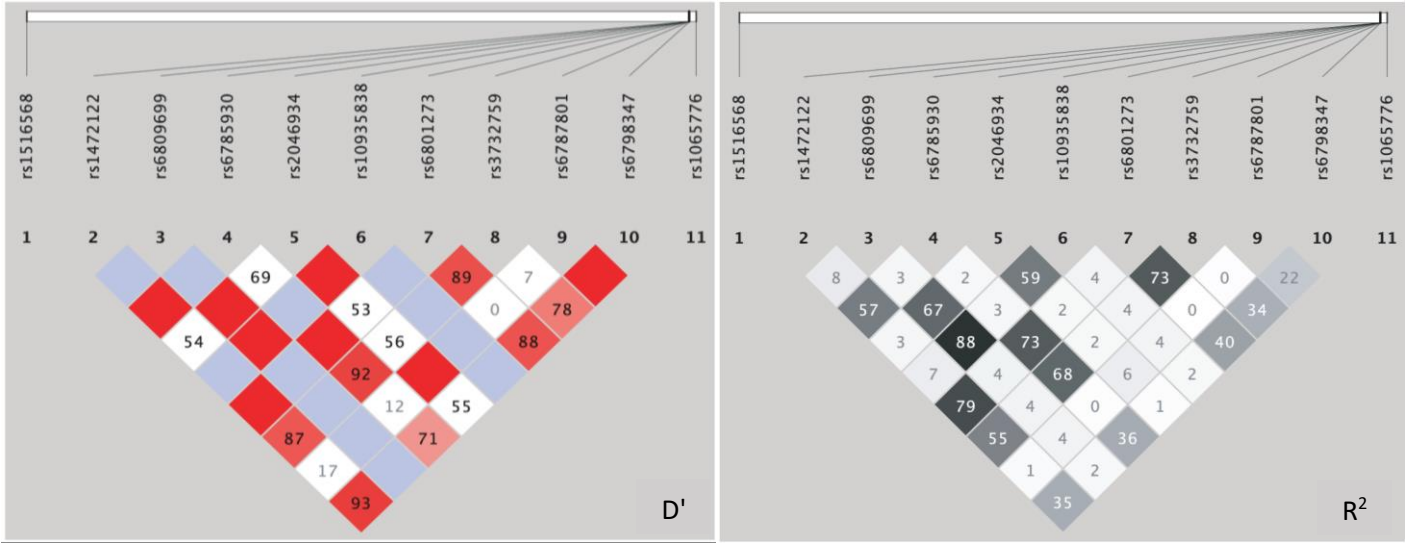

Supplement: S1 Fig — (PDF) [file pone.0306445.s001.pdf]
