# Supplementary material for: The polygenic implication of clopidogrel responsiveness: Insights from platelet reactivity analysis and next-generation sequencing
Source: PLoS One. 2024 Jul 11;19(7):e0306445. doi: 10.1371/journal.pone.0306445 (PMC11239111; doi:10.1371/journal.pone.0306445)

Figure S2. Boxplots - Mean CT values differences respect SNPs

Haplotype 1 Chr 4 - ***NR3C2***

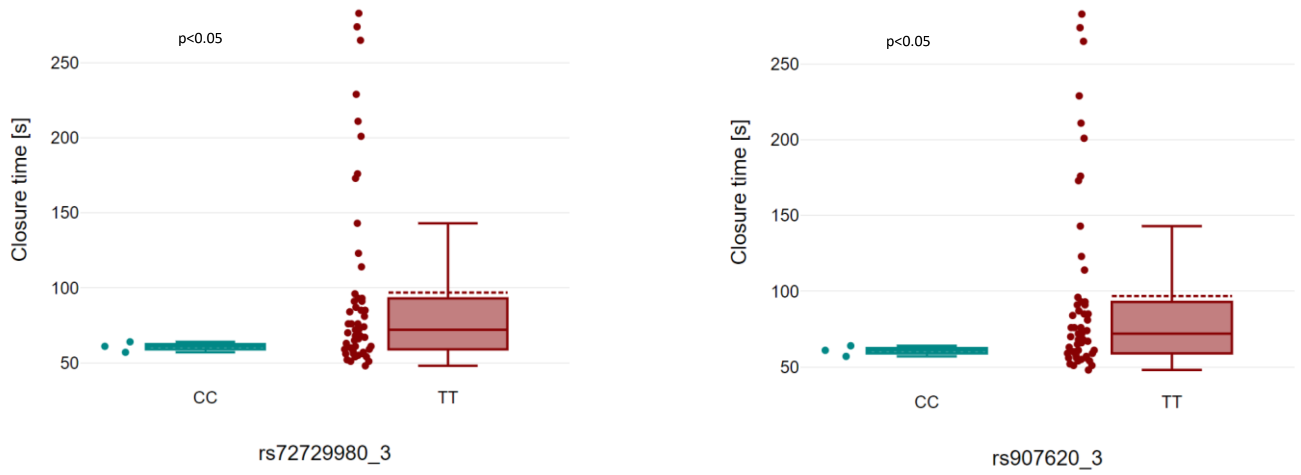

Haplotype 2 Chr 6 - ***PDE10A***

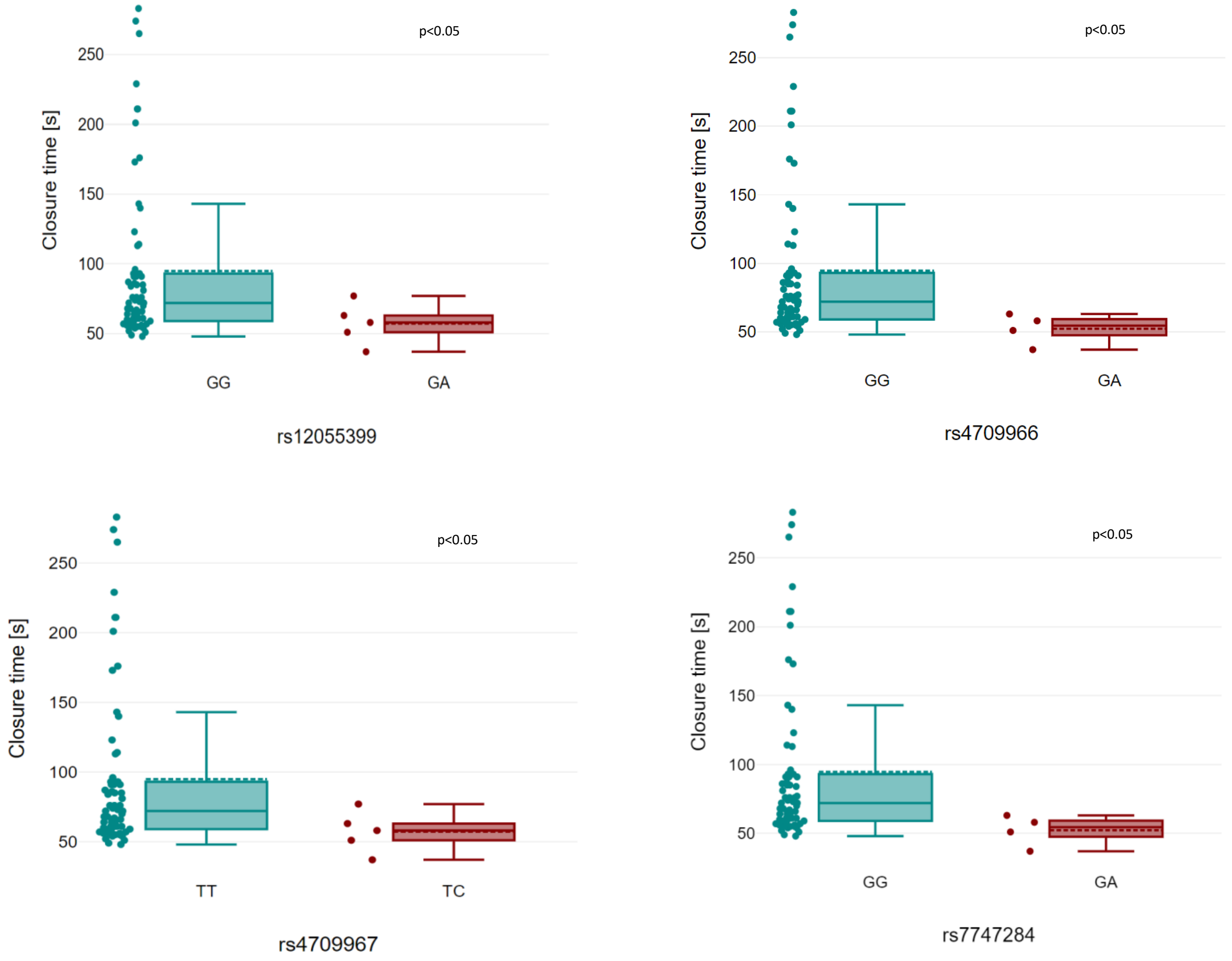

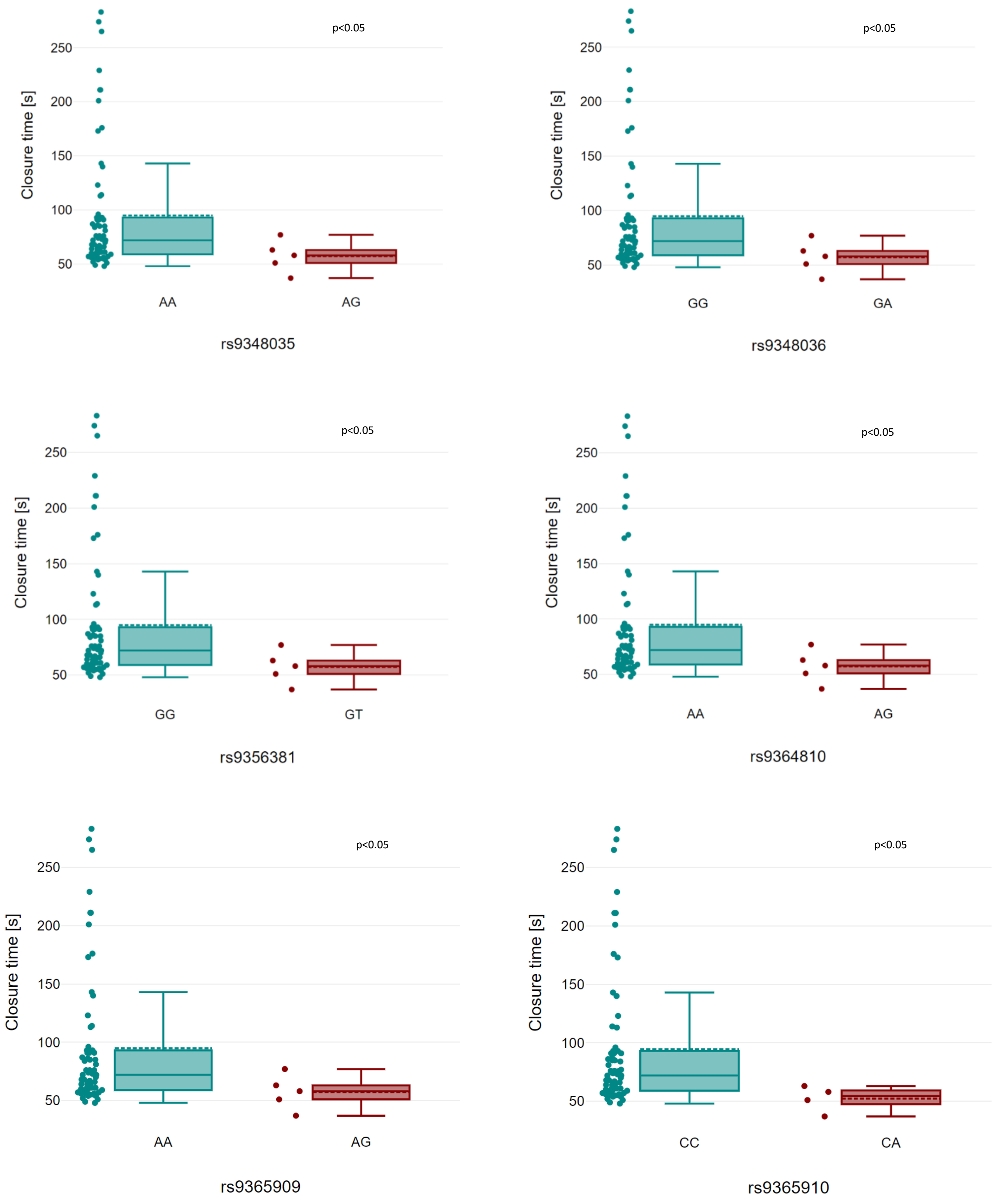
p<0.05
Closure time [s]
250
200
150
100
50
AA
AG
rs9348035
GG
GA
rs9348036
GG
GT
rs9356381
AA
AG
rs9364810
AA
AG
rs9365909
CC
CA
rs9365910

Haplotype 3 Chr 7 – *POR*

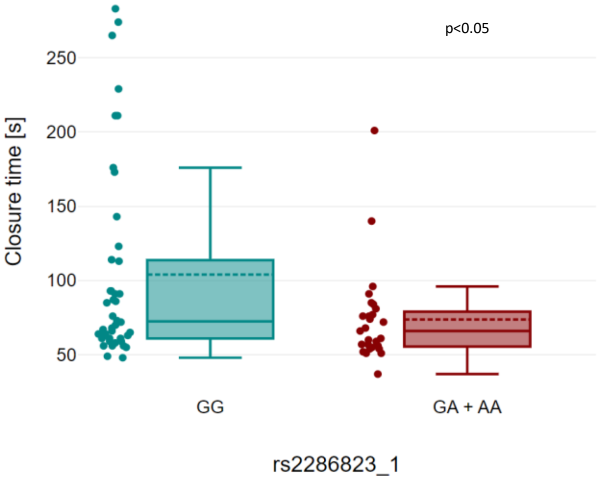

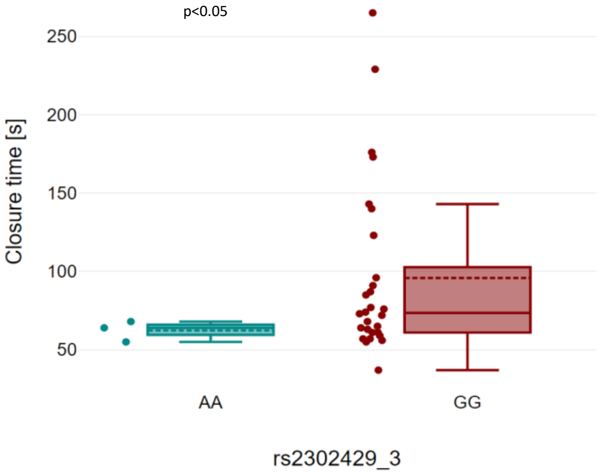

Haplotype 4 Chr 7 - *ABCB1*

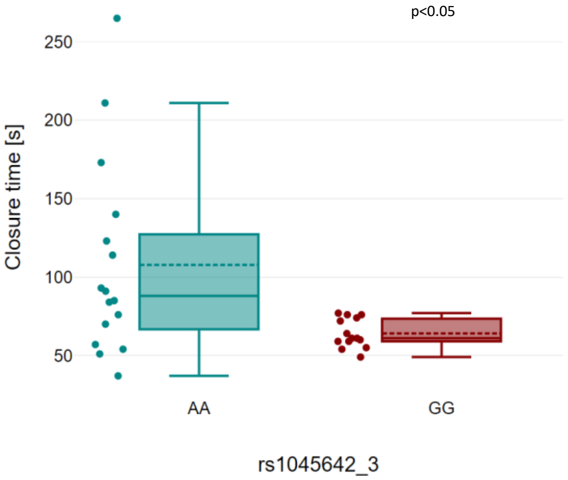

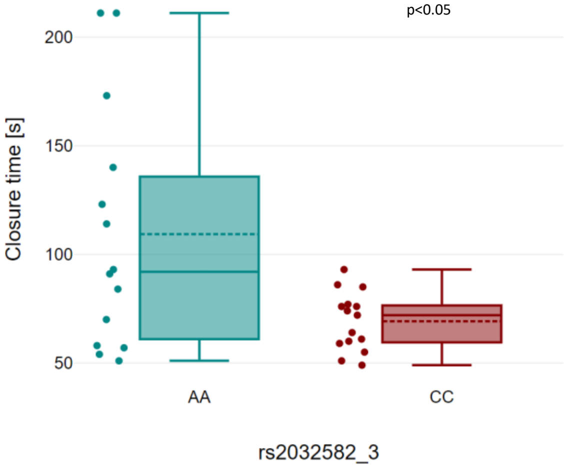

CYP3A5

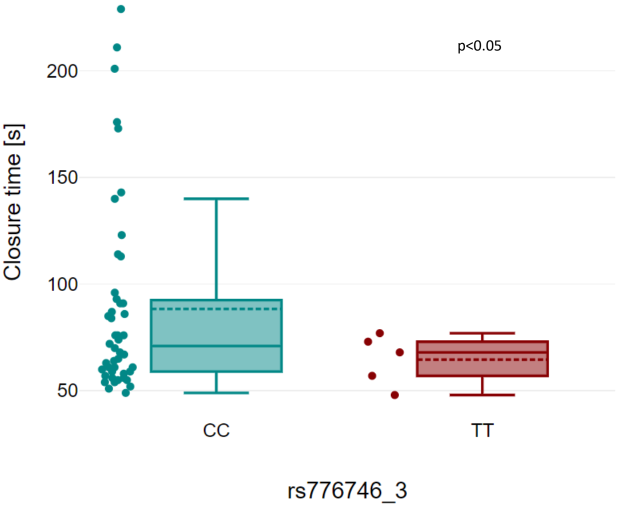

PTGS1

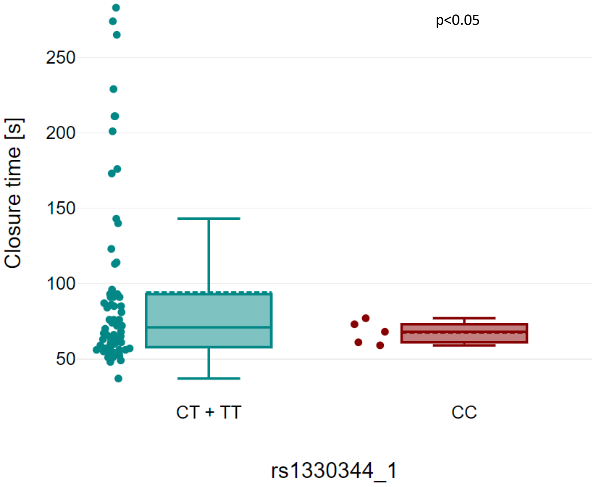

CYP2C19

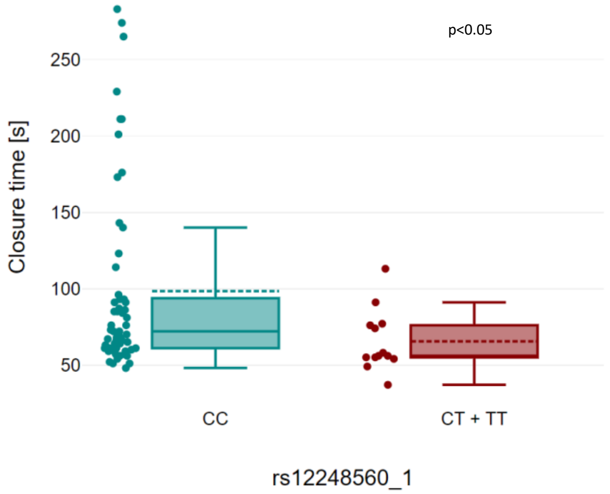

CDH15

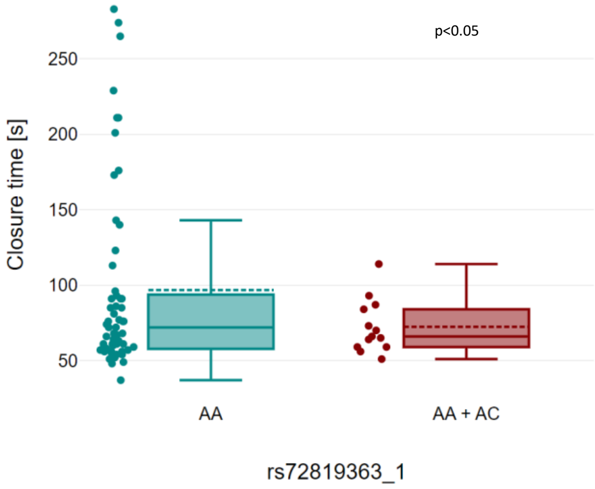

CYP4F2

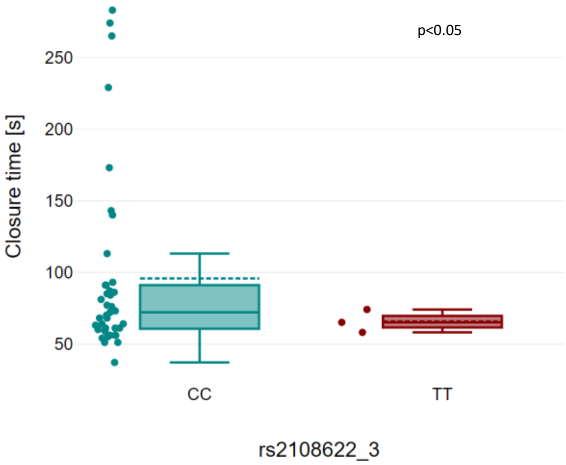

N6AMT1

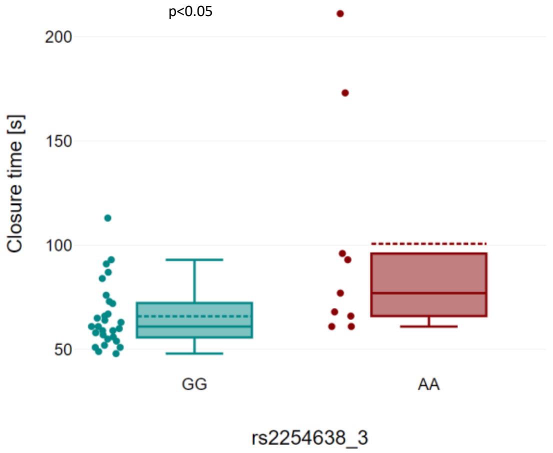

Supplement: S2 Fig — (PDF) [file pone.0306445.s002.pdf]
